# Metabolic Signatures of Extreme Longevity in Northern Italian Centenarians Reveal a Complex Remodeling of Lipids, Amino Acids, and Gut Microbiota Metabolism

**DOI:** 10.1371/journal.pone.0056564

**Published:** 2013-03-06

**Authors:** Sebastiano Collino, Ivan Montoliu, François-Pierre J. Martin, Max Scherer, Daniela Mari, Stefano Salvioli, Laura Bucci, Rita Ostan, Daniela Monti, Elena Biagi, Patrizia Brigidi, Claudio Franceschi, Serge Rezzi

**Affiliations:** 1 Proteomics and Metabonomics, Nestlé Institute of Health Sciences SA, Campus EPFL, Quartier de l'innovation, Lausanne, Switzerland; 2 Applied Mathematics, NESTEC SA, Nestlé Research Center, Lausanne, Switzerland; 3 Department of Medical Sciences, University of Milan, Milan, Italy; 4 Geriatric Unit Ca' Grande Foundation Maggiore Policlinico Hospital, Milan, Italy; 5 Department of Experimental Pathology, University of Bologna, Bologna, Italy; 6 Interdepartmental Centre L. Galvani, University of Bologna, Bologna, Italy; 7 Department of Experimental Pathology and Oncology, University of Florence, Florence, Italy; 8 Department of Pharmaceutical Sciences, University of Bologna, Bologna, Italy; University of Arizona, United States of America

## Abstract

The aging phenotype in humans has been thoroughly studied but a detailed metabolic profiling capable of shading light on the underpinning biological processes of longevity is still missing. Here using a combined metabonomics approach compromising holistic ^1^H-NMR profiling and targeted MS approaches, we report for the first time the metabolic phenotype of longevity in a well characterized human aging cohort compromising mostly female centenarians, elderly, and young individuals. With increasing age, targeted MS profiling of blood serum displayed a marked decrease in tryptophan concentration, while an unique alteration of specific glycerophospholipids and sphingolipids are seen in the longevity phenotype. We hypothesized that the overall lipidome changes specific to longevity putatively reflect centenarians' unique capacity to adapt/respond to the accumulating oxidative and chronic inflammatory conditions characteristic of their extreme aging phenotype. Our data in centenarians support promotion of cellular detoxification mechanisms through specific modulation of the arachidonic acid metabolic cascade as we underpinned increased concentration of 8,9-EpETrE, suggesting enhanced cytochrome P450 (CYP) enzyme activity. Such effective mechanism might result in the activation of an anti-oxidative response, as displayed by decreased circulating levels of 9-HODE and 9-oxoODE, markers of lipid peroxidation and oxidative products of linoleic acid. Lastly, we also revealed that the longevity process deeply affects the structure and composition of the human gut microbiota as shown by the increased extrection of phenylacetylglutamine (PAG) and p-cresol sulfate (PCS) in urine of centenarians. Together, our novel approach in this representative Italian longevity cohort support the hypothesis that a complex remodeling of lipid, amino acid metabolism, and of gut microbiota functionality are key regulatory processes marking exceptional longevity in humans.

## Introduction

The aging phenotype in humans is very heterogeneous and can be described as a complex mosaic resulting from the interaction of a variety of environmental, stochastic and genetic-epigenetic variables [1] .Decades of research on aging have found hundreds of genes [2] and many biological processes [3] that are associated to the aging process, but at the same time, these are still based on few targeted biological outcomes mostly lacking a general molecular footprint which would encompass the longevity process as a as a multi-factorial event [4]. Moreover, the identification of biological markers specific of exceptional longevity is still on its infancy, and their characterization could provide insights into specific molecular mechanisms and/or biological processes of aging. Indeed, aging appears to be characterized by an increasing chronic, low grade inflammatory status indicated as inflamm-aging [5], [6] but centenarians, despite showing some markers of inflammation, avoid or delay the major inflammation-driven age-related diseases, such as cardiovascular disease (CVD), diabetes mellitus (DM) Alzheimer disease (AD), and cancer [7].

Metabonomics is considered today a well-established system approach to characterize the metabolic phenotype, which results from a coordinated physiological response to various intrinsic and extrinsic parameters including environment, drugs, dietary patterns, lifestyle, genetics, and microbiome [8]. Recently, metabonomics had successfully been applied to study the modulation of the aging processes following nutritional interventions, including caloric restriction-induced metabolic changes in mouse [9], dogs [10], and non-human primates [11]. Beyond the insight provided by these studies, a comprehensive metabolic phenotype of longevity in humans has not yet been reported. Using a combined holistic nuclear magnetic resonance (NMR) profiling in urine, targeted liquid chromatography–mass spectrometry (LC-MS/MS) approaches in serum, and a powerful human model of aging and longevity (large group of subjects with an age range of 21–111 years old) objective of this study was to identify the molecular footprints of longevity. Highly informative age groups are represented by centenarians (mean age 100.9 yrs), accepted model of healthy aging and extreme longevity [12], elderly (mean age 70 yrs), and young adults (mean age 31 yrs), all recruited in Northern Italy. Our longevity cohort is composed mostly by females due to reported mortality and genetic characteristics of the Northern Italian population [13], therefore our choice was here to have samples representative of the overall. The elderly individuals were recruited according to strict demographic criteria, an approach which allowed us to further subdivide these subjects having the same chronological age on the basis of their different familial longevity, i.e. offspring of non long-lived parents (mean parental death age, 59.3) and offspring of centenarians, reported to experience a better health and marked delay in the onset of age-related diseases [14]. Moreover, a group of subjects affected by Down 's syndrome (DS, mean age 28 yrs) was studied as a model of premature aging [15] in order to further validate the identified putative metabolic signatures/biomarkers of aging (Figure S1, Text S4, Table S15). Finally, we assessed the associations between changes in bacterial communities' structure and dynamics of host metabolic patterns taking advantage of the fact that a subgroup of young, elderly and centenarians subjects was previously fully characterized for the composition of their intestinal microbiota [16].

## Materials and Methods

### Study Design and Subjects Sampling

Overall a total of 396 subjects belonging to different age groups were enrolled from three Italian cities (Bologna, Milan, and Florence). The group of centenarians consisted of 143 subjects (30 males and 113 females, mean age 100.9±2.1 yrs) born in Italy between the years 1900 and 1908. The elderly group is composed of 210 centenarian's offspring (91 males and 119 females, mean age 70±6.2 yrs) with one centenarian parent born in Italy in the 1900–1908 yrs, and 73 age-matched offspring of non long-lived parents (37 males and 36 females, mean age 70±4.8 years) with both parents born in Italy in the same birth cohort of centenarians but dead before the average life expectancy at 15 years of age (67 years for the father and 72 years for the mother) according to Italian mortality tables. The group of young individuals includes 21 subjects (11 males and 10 females, mean age 30.6±5 yrs). Subjects with Down's syndrome (DS) were also recruited. In particular, a total of 51 DS (26 males, 25 females) of different age, from 12 to 70 years (mean age 28.54±13.0), were enrolled. From a karyotype point of view, 38 were free trisomy, 7 were mosaic, 3 translocations and 3 were diagnosed as DS clinically. The study protocol was approved by the Ethical Committee of Sant'Orsola-Malpighi University Hospital (Bologna, Italy). After obtaining written informed consent, a standard questionnaire (Text S1) to collect demographic data, anthropometric measurements, cognitive and health status, clinical anamnesis, and drug use was administrated to the subjects or to their proxy in case of DS subjects. The health questionnaire was administered by trained personnel and the history of major age-related diseases contributing to morbidity and mortality was accurately reported. Overnight fasting blood samples were obtained in the morning (between 7 and 8 a.m.). Serum was obtained after clotting and centrifugation at 760 g for 20 min at 4°C, and immediately frozen and stored at −80°C. The first morning urine samples were collected in a sterile tube, and immediately stored at −80°C. Full hematochemical and cytokines methods are reported in Supplementary information (Text S2).

### Untargeted serum MS metabonomics

Targeted LC-MS/MS global metabonomic approach on serum samples from the aging cohort (Table 1) was used by combining the Biocrates Life Sciences Absolute*IDQ*™ kit for serum samples and was based on previously published work [17], [18].Well plate preparation and sample application and extraction were carried out according to the manufacturer's instructions. A final volume of 10 µl of serum was loaded onto the provided 96-well plate, containing isotopically labeled internal standards. Liquid chromatography was realized on a Dionex Ultimate 3000 ultra high pressure liquid chromatography (UHPLC) system (Dionex AG, Olten, Switzerland) coupled to a 3200 Q TRAP mass spectrometer (AB Sciex; Foster City, CA, USA) fitted with a TurboV ion source operating in electrospray ionization (ESI) mode. Sample extracts (20 µl) were injected two times (in positive and negative ESI modes) via direct infusion using a gradient flow rate of 0–2.4 min: 30 µl/min, 2.4–2.8 min: 200 µl/min, 2.9–3 min: 30 µl/min. MS source parameters were set at: desolvation temperature (TEM): 200°C, high voltage: −4500 V (ESI−), 5500 V (ESI+), curtain (CUR) and nebuliser (GS1 and GS2) gases: nitrogen; 20, 40, and 50 psi; respectively, nitrogen collision gas pressure: 5 mTorr. MS/MS acquisition was realised in scheduled reaction monitoring (SRM) mode with optimised declustering potential values for the 163 metabolites screened in the assay. Raw data files (Analyst software, version 1.5.1; AB Sciex, Foster City, CA, USA) were imported into the provided analysis software MetIQ to calculate metabolite concentrations.

**Table 1:** Demographic characteristics of the recruited age cohorts.

Metabonomics	Centenerians	Elderly-Offspring of centenarians	Elderly-Offspring of non long-lived parents	Young
*Serum-*Untargeted LC-MS/MS: Gender, *male/female*, Age, years	30/113, 100.9±2 (99–111)	14/32, 68.4±6 (56–81)	19/25, 70.7±6 (59–86)	11/10, 30.±5 (24–40)
*Serum-*Quantitative LC-MS/MS *Eicosanoids:* Gender, *male/female*, Age, years	2/10, 101±2 (99–104)	11/6, 66.3±6 (59–74)	10/10, 73.1±3 (68–76)	9/9, 31.2±5 (25–40)
*Urine-*Untargeted ^1^H-NMR metabonomics: Gender, *male/female*, Age, years	18/74, 100.9±2 (99–111)	91/119, 70.1±6 (55–88)	37/36, 70.3±5 (57–79)	11/10, 30.9±5 (24–40)

Values are presented as mean ±SD with the range in parentheses.

### Targeted serum eicosanoids analysis

A LC-MS/MS method to measure and quantify a panel of 63 inflammatory markers (eicosanoids) was developed in house. Method was based on previously published work [17], [18]. 300 µl of serum samples from remaining available biological material from the three age groups (Table 1) were homogenized with 10 µl of BHT-buffer (butylated hydroxytoluene; 79.2 mg/ml PBS) using the FastPrep® 24 system. 5 µl of the internal standard solution (0.1 ng/µl) is added to 100 µL of plasma sample.10 µl of butylated hydroxytoluene (0.359 µM) is added and the mixture is acidified by adding 15 µl of citric acid (1N). A volume of 550 µl of methanol/ethanol (1∶1, v∶v) was added and samples were mixed during 15 min at 4°C before being centrifuged (3500 rpm, 10 min, 4°C). The organic phase was evaporated to dryness under constant nitrogen flow and the residues were solubilised with 80 µl water, followed by the addition of 20 µL of acetonitrile, before being centrifuged at 3500 rpm for 1 min at 4°C. The supernatant was transferred into LC-MS vials before analysis. Analyses were carried out by liquid chromatography coupled to tandem mass spectrometry (LC-MS/MS). LC was realized on a Dionex Ultimate 3000 ultra pressure liquid chromatography (UPLC) system (Dionex AG, Olten, Switzerland). MS detection was realized on a 5500 Q TRAP mass spectrometer (AB Sciex; Foster City, CA, USA) operating in ESI mode. Gradient chromatographic separation was performed on an Acquity BEH C18 column (2.1×150 mm, 1.7 µm; Waters, Milford, USA). The injection volume was 5 µl and the column was maintained at 50°C. The mobile phase consisted of water containing 1% acetic acid (eluent A) and acetonitrile (eluent B) at a constant flow rate set at 450 µl/min. Gradient elution started from 20% B with a linear increase to 50% B at 6 min, from 50% to 95% B at 13 min, hold for 3 min at 95% B, before going back to 20% B at 16.1 min and reequilibration of the column for additional 11 min. Analytes were monitored in the scheduled selected reaction monitoring (scheduled SRM) mode provided within the Analyst software (version 1.5.1; AB Sciex, Foster City, CA, USA). All mass transitions and MS source parameters are given in supplementary data. The SRM detection window time was set at 120 sec with a target scan time of 0.5 sec. Nitrogen was used as curtain and desolvation gas at the respective pressure of CUR: 20, GS1: 70, GS2: 20 (arbitrary unit). Block source temperature was maintained at 600°C, with the respective voltages: ISV: −4000 V, EP: −10 V, CXP: −5 V. A 15-points calibration curve was realized prior to sample analysis by measuring different dilutions of the standard solution (0–10 ng). Data processing was realized using Analyst software (version 1.5.1; AB Sciex, Foster City, CA, USA). Peak area ratio of each analyte versus its corresponding internal standard or surrogate marker was calculated. It is worth to mention that PGJ2, PGF2a, PGE2, PGE1, 15-oxo-HETE, 15-deoxy-Δ12,14-PGJ2, 6-keto PGF1a, and 5-oxo-ETE were below their detection limit in serum samples and therefore were not taken into account for statistical analysis.

### Annotation of lipid species

LPC, Lysophosphatidylcholines; PC, Phosphatidylcholines; PC-*O*, 1-O-alkyl-2-acylglycerophosphocholines; SM, Sphingomyelines; SM-*OH*, Hydroxy-Sphingomyelin. Individual lipid species were annotated as follows: [lipid class] [total number of carbon atoms]∶[total number of double bonds]. For example, PC 34∶4 reflects a phosphatidylcholine species comprising 34 carbon atoms and 4 double bonds.

### Untargeted urine metabonomics profiling

Urine metabolic profiles were measured on a Bruker Avance III 600 MHz NMR spectrometer equipped with an inverse 5 mm cryogenic probe at 300 K (Bruker Biospin, Rheinstetten, Germany). For each urine ^1^H NMR spectra were registered using pulse sequences including a standard ^1^H detection with water suppression as previously reported [59]–[60]. The peak assignment to specific metabolites was achieved using an internal library of compounds and the literature [61] and confirmed by standard two-dimensional NMR spectroscopy (JRES, TOCSY, HSQC, HMBC) on selected samples. Full method is reported in Supplementary information (Text S3).

### Multivariate Data Analysis

For all the analysis Multivariate Data Analysis (MVA) was performed in several software environments. Thus, data import and pre-processing steps for both ^1^H NMR and targeted MS data were done using ‘in-house’ routines written in MATLAB (version 7.11.0, The Mathworks Inc., Natick, MA, USA). For urine analysis full resolution ^1^H-NMR spectra incorporating data points within the δ 0.4–9.5 region were used for statistical multivariate analysis excluding the water residue signal between δ 4.5–6.5 [19]. In NMR data analysis OPLS-DA models were carried out by using the SIMCA-P+ software (version 12.0, Umetrics AB, Umeå, Sweden). The ^1^H-NMR discriminant model obtained between centenarians and elderly groups (Table S10) generated a model with an AuROC (expressed as area under the ROC curve, AuROC) validation error of 0.93 using again a 13.7% of the total X variance. In NMR data analysis OPLS-DA models (Figure S2) were carried out by using the SIMCA-P+ software (version 12.0, Umetrics AB, Umeå, Sweden). Targeted MS data was analyzed by Random Forests by using the package ‘randomForest’ [20] (http://www.R-project.org/). Spearman autocorrelation matrices were calculated using R and corresponding graphs were produced using the package Rgraphviz v.1.32.0. Univariate significance tests for confirmation were also performed in R.

### Urine metabotypes integration with microbiome data

The fecal microbiota of a randomly selected number of individuals from the aging cohort (16 centenarians, 21 elderly and 19 young) was characterized by HITChips as previously published [16].

## Results

### Clinical Characteristics of the age cohort

We performed targeted liquid chromatography–mass spectrometry (LC-MS/MS) in serum and nuclear magnetic resonance (^1^H-NMR) profiling in urine in human model of aging and longevity, compromising young, elderly, and centenarian individuals (Table 1). Cohort sampling was based on availability of biofluid samples (urine) and availability of remaining (serum) samples from different targeted LC-MS/MS profiling applied methods assuring proper uniform distribution among genders (elderly) and parental longevity (centenarians' offspring and offspring of not long living parents). As expected, centenarians were characterized by a number of significant differences regarding a variety of parameters (Table S1, BMI p<0.001, HOMA p<0.001, total cholesterol p<0.01, triglycerides p<0.05, HDL p<0.01, LDL p<0.01, A-SAA p<0.001, and CRP p<0.001 among others) in comparison to elderly (including offspring of centenarians and offspring of non long-lived parents) and young subjects. In addition, centenarians showed low prevalence of severe cognitive decline, as measured by the Mini-Mental State Examination test (MMSE) [21].

### Serum lipidome signatures of aging and longevity

**Figure 1:**
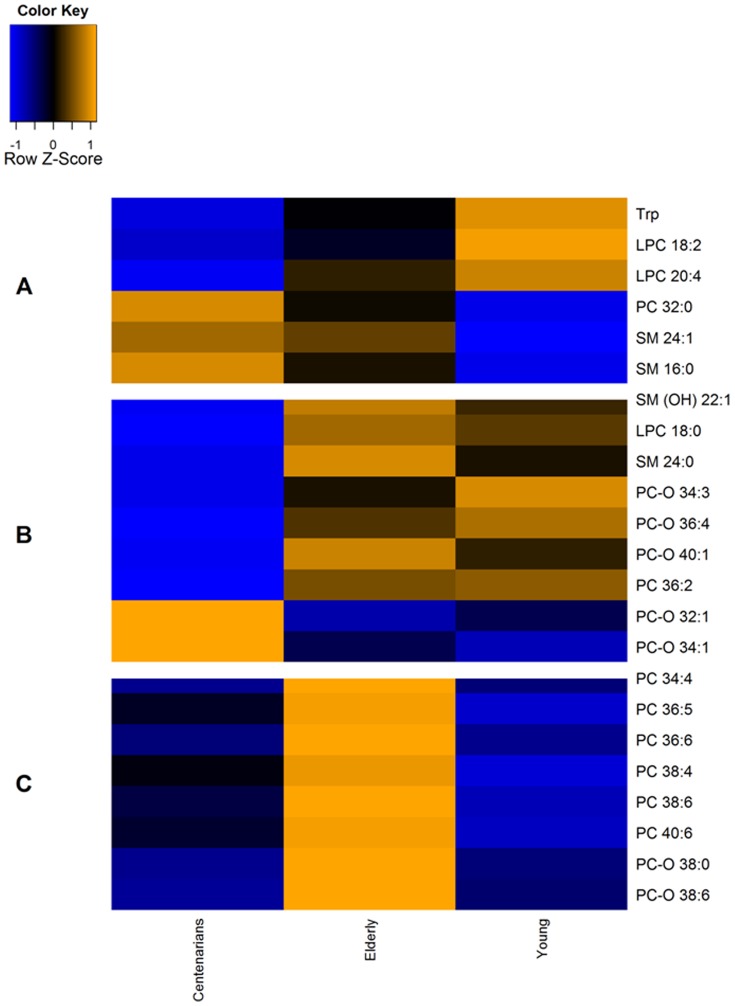
Metabolic signature of aging and longevity in serum. Targeted LC/MS metabonomics on the aging cohort (young, elderly, and centenarians) revealed three trends within the identified markers. (A) Set of metabolites that decreased/increased within age. (B) Set of metabolites that deceased or increased in centenarians only. (C) Set of metabolites maintained at similar concentration level in centenarians and young people but not in the elderly. Reported is median value in µM among the three age groups. Blue denotes negative/decreased concentration, orange denotes positive/increased correlation, black denotes no changes. All significantly regulated metabolites are listed in Table S3.

To unravel comprehensive metabolic signatures of aging we implemented targeted LC-MS/MS global metabonomic approach on serum samples from 143 centenarians, 90 elderly and 21 young individuals (Table 1). We performed multivariate data analysis using Random Forest (RF™) on pre processed semi-quantitative data on the given 160 metabolites (amino acids, sugars, acyl-carnitines, sphingolipids, and glycerophospholipids). Using the variable importance feature implemented in RF™, we uncovered the metabolic signatures that better discriminates among the three age groups. To assess the individual discriminant ability of each component of the signature, we applied Wilcoxon Rank sum tests among the age groups. While the overall concentration of glycerolphospholipids and sphingolipids increased and decreased depending on the fatty acid composition, we identified three distinct metabolic patterns : (i) set of compounds that that monotonically increased or decreased (statistically significant) with age (Fig. 1A, Table S3), likely representing metabolic signatures of the aging process, such as decreased concentrations of Tryptophan (Trp), lysophospatidylcholines (LPC 18∶2, LPC 20∶4), increased levels of PC 32∶0 and sphingomyelins (SM 24∶1, SM 16∶0); (ii) a set of compounds remaining largely unchanged until age 70 and undergoing significant changes in centenarians (Fig. 1B, Table S3), characterized by a complex pattern of decreased concentration in sphingomyelins and specific glycerophospholipids (SM-*OH* 22∶1, LPC 18∶0, SM 24∶0, PC-*O* 34∶3, PC-*O* 36∶4, PC-*O* 40∶1, PC 36∶2) and increased concentration in specific glycerophospholipids (PC-*O* 32∶1, PC-*O* 34∶1); (iii) set of compounds which changes in the elderly, but is remarkably similar in young subjects and centenarians, (Fig. 1C, Table S3), putatively representing the metabolic phenotype of longevity, characterized by an increased concentration of specific glycerophospholipids (PC34∶4, PC36∶6, PC 36∶5, PC 38∶4, PC 38∶6, PC 40∶6, PC-*O* 38∶0, PC-*O* 38∶6).

**Figure 2:**
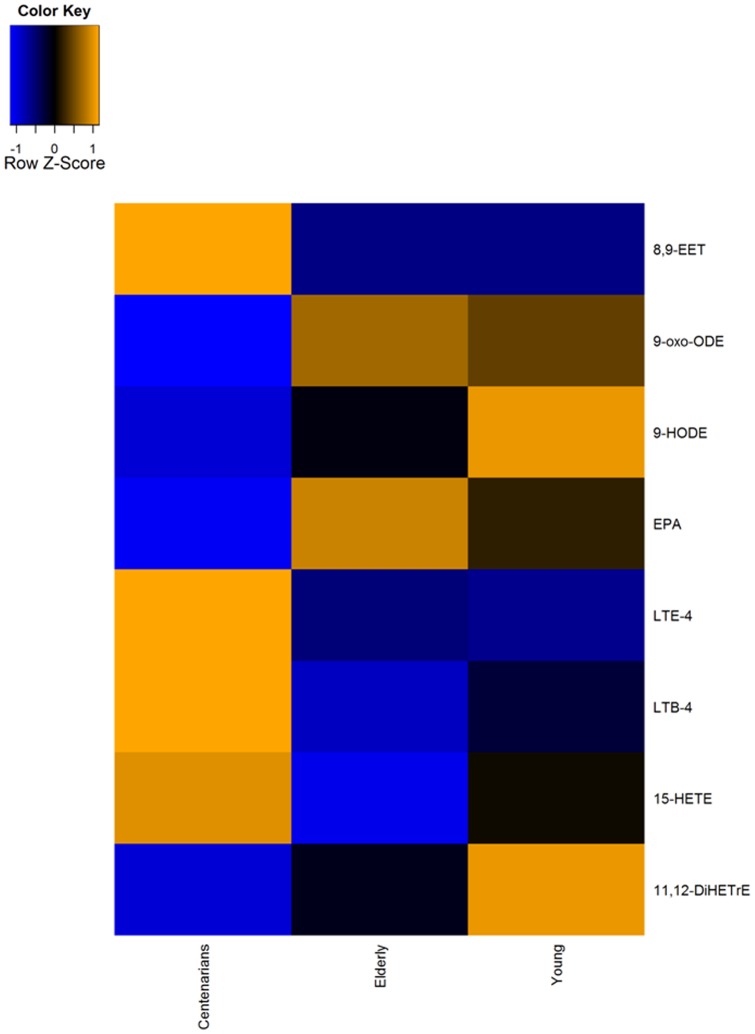
Metabolic signature of aging and longevity in serum as per LC/MS eicosanoids profiling. Reported is median value in ng/100 µl serum among the three age groups. Blue denotes negative/decreased concentration, orange denotes positive/increased correlation, black denotes no changes. All significantly regulated metabolites are listed in Table S6.

To further investigate the centenarian's response to immune and inflammatory processes we performed a quantitative LC-MS/MS eicosanoids profiling on serum samples from a restricted number of 12 centenarians, 37 elderly and 18 young subjects (Table 1). Compared to elderly and young individuals, RF™ on quantitative data displayed statistical changes in the centenarian group, (Fig. 2) as assessed by Wilcoxon rank sum test (all significantly regulated metabolites are listed in Table S6). Specifically, centenarians exhibit lower concentration of 11,12-dihydroxy-eicosatrienoic acid (11,12-DiHETrE), 9-hydroxy-octadecadienoic acid (9-HODE), and 9-oxo-octadecadienoic acid (9-oxo-HODE), and increased concentrations of 15-hydroxy-eicosatetraenoic acid (15-HETE), and leukotriene E4 (LTE4). Compared to elderly levels of eicosapentaenoic acid (EPA) decreased also in centenarians. Furthermore, to maximize metabolic changes between centenarians and elderly, we applied pair-wise Multiple Regression/Correlation (MRC) analysis between these two age groups displaying increased serum concentration levels of 8,9-epoxyeicosatrienoic (8,9-EET) in centenarians.

Lastly, we have reported concentration values for the selected metabolites of interest for female and male individuals in both global metabonomic and eicosanoids analysis. For global metabonomic profiling no differences were displayed for selected metabolites after gender separation (Tables S4, S5). For targeted eicosanoids analysis the majority of samples represent females individuals (Table 1), therefore the displayed statistical differences remain when looking only in females (Tables S7), while in males, as the number among centenarians is very low, and we report their values with the overall metabolic changes kept (Tables S8), it is worth to mention that this leads to limited statistical power.

### Metabolic phenotypic differences within the elderly group according to parental longevity

**Figure 3:**
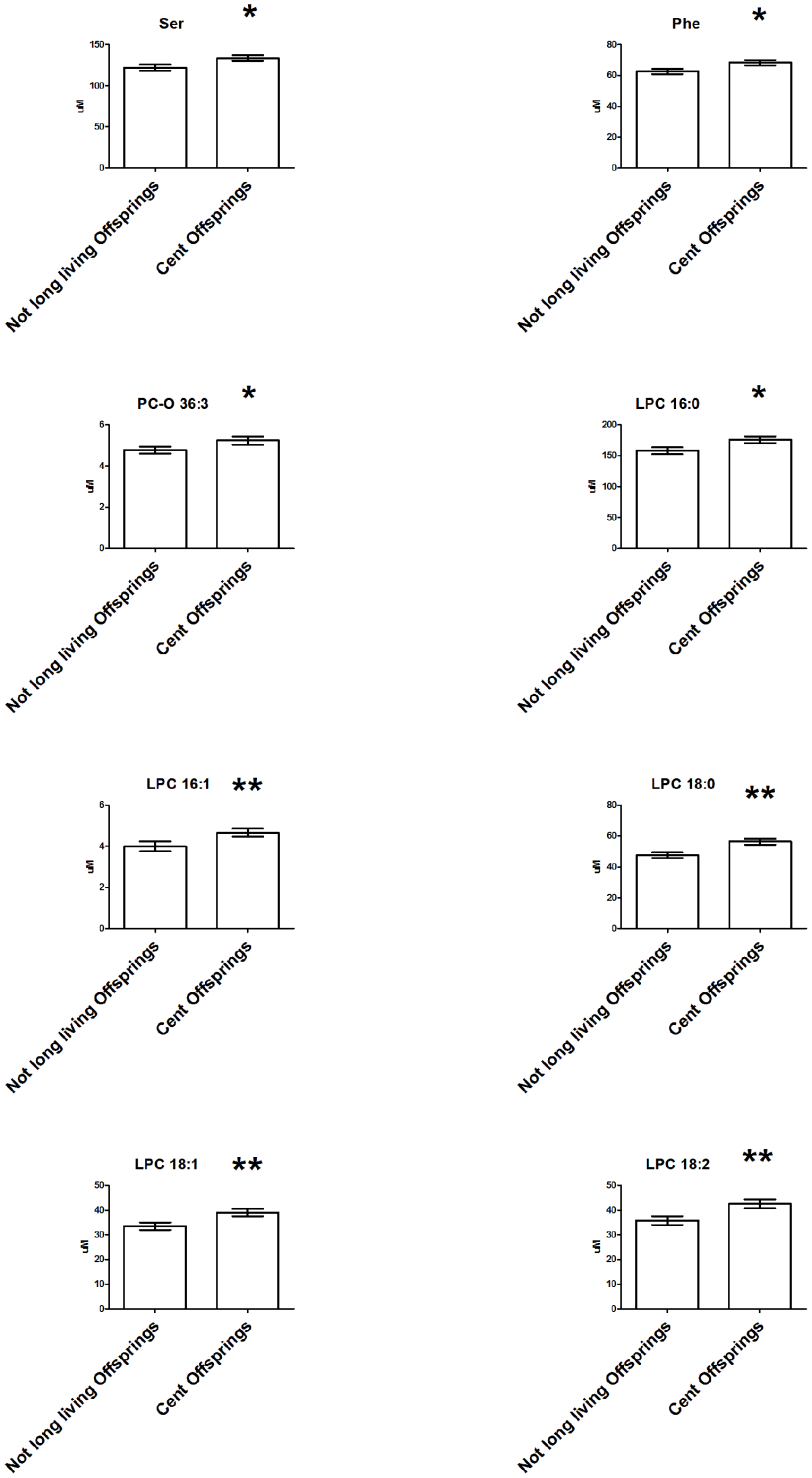
Differences in metabolic profiles as displayed by LC/MS-MS targeted approach between centenarian's offspring (46 subjects average age 68.4 yrs) and offspring of non long-lived parents (42 subjects average age 70.7 yrs). Bar plots indicating mean (µM) ±standard error. All significantly regulated metabolites and statistical changes are listed in Table S9. Significant differences were assessed by Mann-Whitney U test where *p<0.05., **p<0.01, ***p<0.001.

To identify metabolomic biomarkers capable of distinguishing between the two subgroups (elderly offspring of centenarians and elderly offspring of non long-lived parents), we further analyzed by RF™ the group of elderly taking into account the age of their parents (familial longevity, Table S2). Here, we underpinned in centenarian's offspring (46 subjects, average age 68.4), compared to offspring of non long-lived parents (44 subjects, average age 70.5) (Figure 3, Table S9), higher concentrations of specific lysophospatidylcholines (LPC 16∶0, LPC 16∶1, LPC 18∶0, LPC 18∶1, LPC 18∶2), glycerophospholipids (PC-O 36∶3) and two amino acids (serine, phenylalanine). In the measured eicosanoids panel, no metabolic differences were noted among centenarians' offspring (17 subjects, average age 66.3) and offspring of non long-lived parents (20 subjects, average age 73.1). No metabolic differences in urine profiling were discerned among centenarian's offspring (210 subjects, average age 70.1) and offspring of non long-lived parents (73 subjects, average age 70.3).

### Metabolic signature of longevity in ^1^H-NMR urine profiles mirroring changes in centenarians gut microbiota

**Figure 4:**
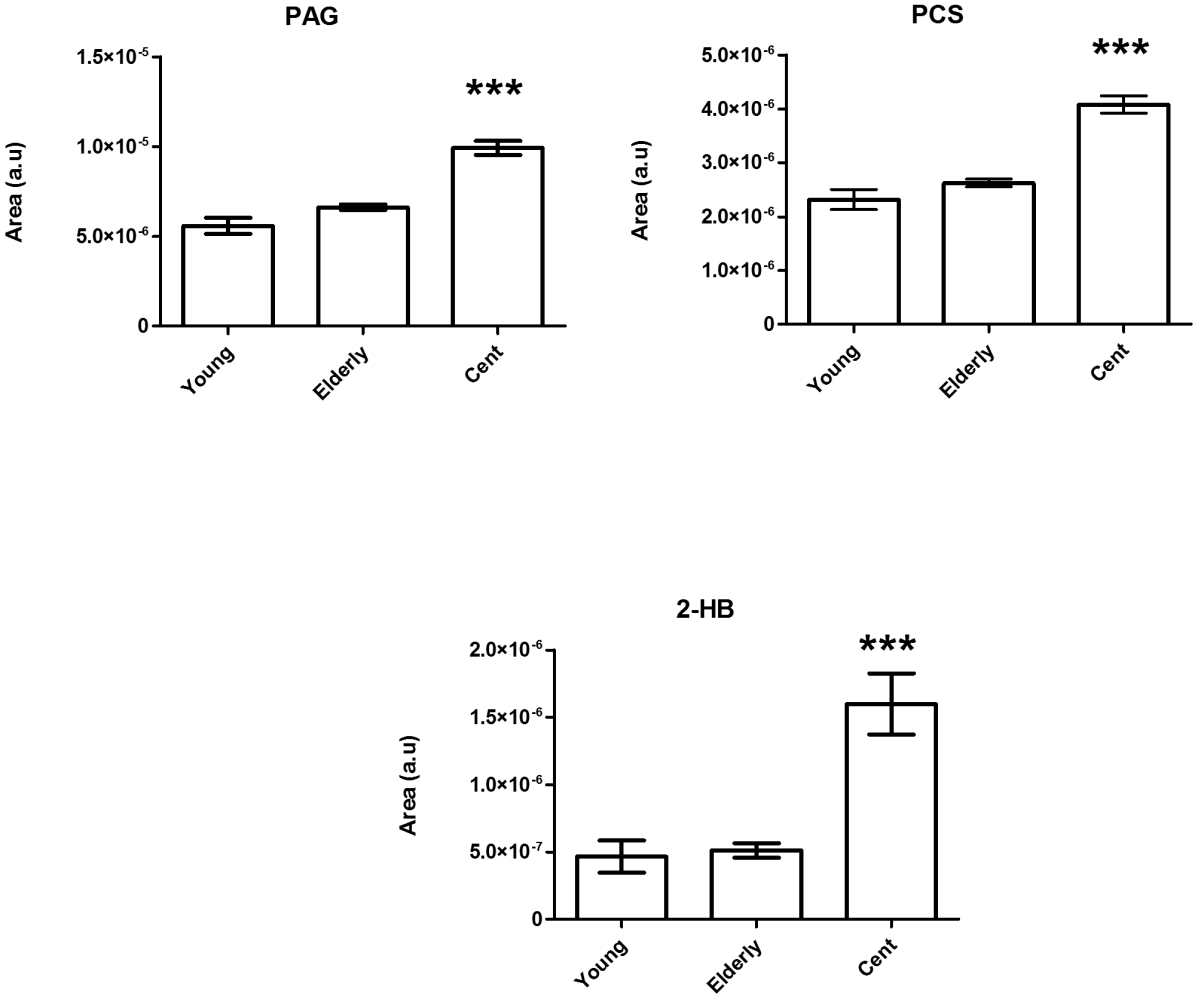
Markers of longevity as per ^1^H-NMR urine profiling. Bar plots indicating mean (relative concentration) ±standard error. PAG = Phenylacetylglutamine, PCS = p-cresol-sulfate, 2HB = 2-hydroxybenzoate. All significantly regulated metabolites and statistical changes are listed in Table S12. Significant differences were assessed by Mann-Whitney U test where ***p<0.001.

To underpin longevity-induced changes in urine we performed 600 MHz ^1^H-NMR metabolic profiling on the three age-groups (Table 1, 92 centenarians, 283 elderly and 21 young adults). We processed the data by multivariate analysis while we applied Orthogonal Projection on Latent Structures – Discriminant Analysis (OPLS-DA) (Fig. S11A) on unit variance scaled data. Interpretation of the O-PLS-DA regression coefficients (Figure S2B) for the first latent component [19], displayed higher levels of phenylacetylglutamine (PAG), p-cresol-sulfate (PCS), and 2-Hydroxybenzoate (2-HB) in centenarians compared to elderly. To gain semi-quantitative information, peak areas in the original spectra were integrated for these three metabolites and differences with statistical significance were confirmed by using Wilcoxon Rank Sum test (Figure 4, Table S11). Lastly, we have reported values for three metabolites of interests in urine in females and males individuals, displaying no gender differences for PAG, PCS, 2-HB (Tables S12, S13).

## Discussion

In the present study we have characterized for the first time, by using a complementary NMR and MS-based metabonomics and lipidomic approach, in both serum and urine, the metabolic phenotype (metabotype) of extreme longevity in a representative Northern Italian population composed mostly by female individuals. While the young individuals are limited in numbers they serve, from an observational point of view, as a representative aging group to distinguish specific metabolic changes delayed or preserved during the ageing of centenarians, from metabolic features that are either a continuation of normal ageing or indicative of a drift in the ageing processes. Comprehensive MS-based targeted metabonomics serum analysis revealed important biological changes associated to aging (Fig. 1, Table S3). Among these changes we reveal an age-related reduction of Tryptophan (Trp) concentration, supporting the proposed link among its decreased level and the raise of chronic low-grade inflammatory conditions [22].Several studies found patients with inflammatory diseases to have significant elevations in serum kynurenine and depletion of Trp compared to control population [23]. Recent work also displayed the relationship between reduced serum Trp and increase immune activation [24]. With increasing age we observed lower concentration of lysophospatidylcholines (LPC 18∶2, LPC 20∶4). While LPCs exhibit different physical and biological properties based on fatty acid chain length and degree of unsaturation, phospholipids are inflammatory mediators [25], with atherogenic properties [26] and their altered levels are linked to age-related physiological changes [27]. Lastly, with increasing age we found an increase in SM 24∶1 and SM 16∶0. Consistent with our findings, alteration of plasma lipid profiles were previously observed in different aging and caloric restriction animal models [11], [28].

Interestingly, the metabolic differences seen in the elderly cohort support the notion that siblings of longevity parents have a distinctive aging metabolic phenotype from their age matched controls [29] (Figure 3, Table S9). While the exact biological significance of the noted LPCs and amino acids changes, and how these might be related to longer life expectancy and/or delay in age-related diseases, are not clear at the moment, and worth further investigation, it is crucial to denote that serine is needed for the metabolism of fats and fatty acids, muscle growth, and to maintain a healthy immune system and was previously found to decrease in plasma under inflammatory conditions [30], while phenylalanine exhibits anti-inflammatory properties and it is often used to treat arthritis and Parkinson's disease [31].

Further, our study reveals that longevity can be ascribed as a distinct metabolic phenotype marked by specific changes in serum lipid profile (Fig. 1A, 1C, Table S3). Centenarians are characterized by decreased concentrations of the sphingomyeline SM 24∶0 and SM-OH 22∶1, and the diacylphosphatidylcholine PC 36∶2. SMs species are important cellular membrane constituents which are tightly associated with cholesterol in construction, metabolism and transport, and which are enriched in lipid rafts. The physiological role of SM is still unclear and previous studies report diverging hypothesis on their relationship with cardiovascular risk conditions [32], [33]. Centenarians exhibited also specific monotonic changes in concentration of LPC 18∶0, while varying in concentrations of several acyl-ether, PC-O species (decreased PC-*O* 34∶3, PC-*O* 36∶4, PC-*O* 40∶1, and increased PC-*O* 32∶1, PC-*O* 34∶1). Plasmalogens containing a vinyl ether bond link to the *sn*-1 aliphatic chain of the glycerol backbone are endogenous antioxidant. Several studies have indicated that plasmalogens are depleted in a number of human pathologies with their levels changing in response to oxidative damage [34].

We also uncovered a set of compounds remarkably similar in circulating levels among young subjects and centenarians, while varying in the elderly only, characterized by an increased concentration of specific glycerophospholipids mostly polyunsaturated diacyl phospholipids (PC) (Fig. 2C, Table S3). PC is a major structural lipid of the cell membrane and it is involved in lipid metabolism being crucial for lipid transport. Interestingly, increased concentrations of plasma polyunsaturated fatty acids have been implicated in the pathogenesis of chronic diseases [35]. At the moment we do not know how modification of cell membrane lipid composition leads to functional changes in longevity metabolic processes, including response to chronic diseases. If replicated in larger studies, the altered metabolites might be considered as potential biomarkers in the generation of new hypotheses on the biological mechanisms behind longevity. Yet, although our observational data cannot drive any concluding statements on the possible causality linkage between inflammatory status, aging, and modulation of lipid metabolism, it is nowadays well accepted that the down-regulation of the mammalian target of rapamycin (mTOR) signaling pathway is a central regulatory process of pro-longevity in mammals [36]. The mTOR encompasses a series of regulatory multi-protein complexes from the the kinase family involved in cellular response to multiple triggers including nutrients, and reactive oxygen species (ROS), and trough the mTORcomplex 1 (mTORC1) possibly controlling lipid biosynthesis [37]. The down-regulation of this pathway has resulted in observing an extension of lifespan in multiple organisms [38]. The regulation of mTOR also cross-talks with AMP-activated kinase, Sirt1, and Insulin/IGF-1 in a concerted manner that points to consider this complex network as a model of longevity [39]. The down-regulation of mTOR can activate Sirt1 and the AMPK pathways, and expression of specific mitochondrial genes and fatty acid oxidation. One could hypothesize that the downstream effect of these complex intracellular processes would lead to the expression of a specific blood lipid profile with a plausible remodeling on the composition of phospho/sphingolipids and lipoprotein particles specific to the centenarian phenotype. Indeed correlation analyses (Table S14) display that the LDL concentration is correlated to specific SMs (SM 16∶0 r^2^ = 0.34, SM-OH 22∶1 r^2^ = 0.40, SM 24∶0 r^2^ = 0.39), while PC-O species (PC∶O 34∶3 r^2^ = 0.57, PC∶O 38∶0 r^2^ = 0.38, PC∶O 38∶6 r^2^ = 0.39) are positively correlated to the HDL circulating level, a feature previously reported for ageing [40]. Thus it appears also that the displayed differences in concentration levels of PC-O species in centenarians might be due to variation in coping ROS, while supporting their role as serum antioxidants preventing lipoprotein oxidation.

Furthermore, we depicted specific changes in the longevity phenotype in arachidonic acid synthesis (Fig. 2, Table S6), key mediator of immune and inflammatory reactions. Here we discerned higher concentration of leukotriene 4 (LTE-4), which plays a pivotal role in allergic and inflammatory diseases, causing increased vascular permeability and vasodilatation [41]. Centenarians displayed higher circulating levels of 15-hydroxy-eicosatetraenoic acid (15-HETE), a major product of 15-lipoxygenase (15-LOX) enzyme, known for its anti-inflammatory properties [42]. Increased activation of cytochrome P450 pathway in centenarians is further supported by increased circulating levels of 8,9-epoxyeicosatrienoic (8,9-EpETrE) and decreased concentration of 11,12-dihydroxy-eicosatrienoic acid (11,12-DiHETrE). EpETrE are important components of many intracellular signaling in both cardiac and extracardiac tissues [43]. EETs display anti-inflammatory effects by inhibiting nuclear factor kappa B (NF-κB)-mediated gene transcription [43], [44]. EpETrEs can be further metabolized by soluble epoxide hydrolase (sEH) to dihydroxy-eicosatrienoic acids (DiHETrE), reducing their original biological activity. Therefore, the decreased concentration of 11,12-DiHETrE might reveal decreased sEH's effect on its precursor 11,12-EpETrE. Most important, centenarians displayed lower circulating levels of 9-hydroxy-octadecadienoic acid (9-HODE), a biological active molecule, marker of lipid peroxidation, and in 9-oxo-octadecadienoic acid (9-oxo-HODE), a stable oxidation product of linoleic acid, the generations of which is increased with increasing oxidative stress [45]. Previous studies determined the positive relationship among lower 9-HODE concentrations and Mediterranean diet to reduced cardiovascular disease risk [46], and our results might underpin this trend, while at the same time revealing decreased oxidative damage in centenarians. Increased levels of lipid oxidation products such as 9-oxoODE are normally detected in plasma samples of patients suffering rheumatoid arthritis [47], and arthrosclerosis [48]. Interestingly, regulatory process involving activation of cellular detoxification, through the nuclear erythroid 2-related factor (Nrf2) signaling pathway, is also being proposed as an element of increased lifespan [49]. Our findings on the increased concentration of 8,9-EpETrE, suggesting increased activity of CYP enzyme, would support promotion of cellular detoxification mechanisms through specific modulation of the arachidonic acid metabolic cascade in centenarians. Such effective mechanism might results in the activation and successful antioxidative response, as displayed by the decreased concentration of 9-HODE, and 9-oxoODE in centenarians. Compared to elderly, centenarians also presented depletion of eicosapentanoic acid (EPA), an omega-3 fatty acid, which can be synthesized in humans from alpha-linoleic acid or in greater amount directly from oily fishes. Importantly, EPA exerts anti-inflammatory effects mostly by increasing the biosynthesis of beneficial ω-3 eicosanoids, resolvins [50].

Taken all together, we propose that the overall lipidome changes, above elucidated, might putatively reflect centenarians' unique capability to adapt/respond to the accumulating oxidative and chronic inflammatory processes characteristics of their extreme aging phenotype.

**Figure 5:**
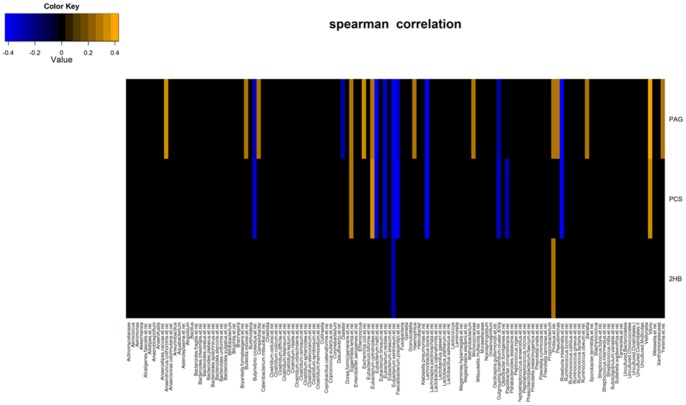
Spearman correlation map between urine markers of longevity (PAG = phenylacetylglutamine, PCS = p-cresol sulfate, 3-HB = 3-hydroxybenzoate) and order/genus-like bacterial phylogroups. Blue denotes negative correlation, orange denotes positive correlation, and black denotes no correlation.

In the elderly population associations among gut microbiota and inflammatory status had been clearly displayed [51], [52]. In our study urine metabolic profiling revealed that the longevity phenotype is affected by significant changes in the gut microbiome as displayed by increased excretion of PCS and PAG (Fig. 4, Table S11) in centenarians compared to elderly. Our findings support the initial hypothesis suggesting that late aging process might lead to increase *p*-cresol production, driven by age-related changes in the composition of the gut bacteria [53]. Gut microbiota extensively catabolized protein and aromatic amino acids, including phenylalanine and tyrosine, to form PAG and PCS [54]. Moreover, centenarians display increased concentration of 2-HB (Figure 4) a compound present in most fruits and vegetables, with anti-inflammatory activity and with known capabilities to inhibit transcription of cyclooxygenase-2, an enzyme that catalyses the formation of prostaglandins during inflammation [55]. Lastly, we assessed the relationships between urine metabotypes and microbiota composition correlating the three markers of longevity (PAG, PCS, 2-HB) with phylogenetic bacterial groups (Fig. 5). PAG displays positive correlation with Proteobacteria species, namely *Campylobacter*, *E. coli*, *Haemophilus*, *Pseudomonas*, *Serratia*, *Yersinia et rel*, while both PCS and PAG correlating to *Vibrio*. Although it has been reported that some species of Clostridia produce phenol and p-cresol together with ammonia and hydrogen by anaerobic degradation of aromatic amino acids [56], our data suggest that Proteobacteria might also contribute to the pool of PAG and PCS. Previous studies have shown that *E. coli* isolated from Crohn's Disease patients have pathogen-like behavior in vitro, and may play a role in the inflammatory process [57]. Of particular interest is the negative correlation of PAG and PCS with several butyrate-producing bacteria belonging to Clostridium cluster XIVa species, namely *Butyrivibrio crossotus et rel.*, *E. hallii*, *E. rectale*, *E. ventriosum*, *F. prausnitzii*, *Roseburia intestinalis* as it is demonstrated that butyrate, a short chain fatty acid mainly produced in the gut by Firmicutes of Clostridium clusters IV and XIVa, has a protective role against chronic inflammatory diseases [58]. Of interest is also the positive correlation of 2-HB with *Proteus et. rel*. Taken together these data display that the longevity process deeply affects the structure and composition of the human gut microbiota, with centenarians displaying lower contribution of Clostridium cluster XIVa, and relatives symbiotic species with reported anti-inflammatory properties, and relative increase of facultative anaerobes including Proteobacteria [16].

It is imperative to note that while this study portrays a sampling representative of a limited geographic area (northern Italy), where dietary and lifestyle factors can be assumed similar, our newly reported longevity metabolic footprint needs further validation across populations with different genetic backgrounds, environmental conditions, and increased representation of both the genders. While it is difficult to recruit a larger number of male centenarians, due to significant acknowledged differences in male to females ratio distribution in the north of Italy [12], we have reported values for selected metabolites after gender separation which provides a first insight into the displayed metabolites of interest. Here, whilst the displayed metabolic differences of longevity are maintained, after gender separation, our findings will be compared and validated in much bigger cohorts to provide future predictive utility, such as the ongoing EU Mark-Age (http://edukon.biologie.uni-konstanz.de/mark-age). Further future studies will also have to address how nutritional challenges and different dietary regimes in human aging populations impact these biomarkers confirming the observed changes in the discussed metabolic pathways.

In conclusion, our study contributes to shed light onto the complex mosaic of human aging and longevity providing comprehensive metabolic outcomes in biofluids that can be paralleled with the currently investigated mechanistic routes of the mTOR, AMPK, inflammation, and changes in the gut microbiota, underlining the expression of human longevity phenotype. As centenarians well represent the model of healthy and successful aging, there are many important implications in understanding the underlying molecular mechanisms behind such acquired longevity and how environmental factors and nutrition might aid in shaping such acquired successful metabolic phenotype.

## Supporting Information

Figure S1 **Validation of metabolic signature of biological aging in serum of DS individuals.** Bar plots representing mean ±standard error. Despite their young age (mean age 28 yrs), concentration of Trp, LPC 18∶2, and LCP 20∶4 is closer to levels seen in centenarians (LPC 20∶4) and elderly (Trp, LPC 18∶2). All significantly regulated metabolites and statistical changes are listed in Table S4. Significant differences were assessed by Mann-Whitney U test where *p<0.05., **p<0.01, ***p<0.001.
(TIF)

Figure S2 **OPLS-DA score (A) and coefficient plots (B) derived from urinary ^1^H-NMR spectra from elderly (blue) and centenarians (red).** PAG = phenylacetylglutamine, PCS = p-cresol sulfate, 3-HB = 3-hydroxybenzoate.
(TIFF)

Table S1 **Demographic, anthropometric, clinical, and haematochemical characteristics of the recruited age groups.** Values are presented as mean ^±^SD with the range in parentheses. Significant differences were assessed by Mann-Whitney U test where “a” refers to differences between centenarians' and elderly and “b”' refers to differences between elderly and young and marked as follows: *p<0.05., **p<0.01, ***p<0.001.
(DOCX)

Table S2 **Demographic, anthropometric, clinical and haematochemical characteristics of the elderly cohort analyzed by metabolomic targeted MS.** Values are presented as mean ±SD with the range in parentheses. Significant differences were assessed by Mann-Whitney U test where no statistical differences are reported between centenarians' offspring and offspring of non long-lived parents.
(DOCX)

Table S3 **All significantly regulated metabolites in blood serum (mean values ± SD) from the targeted MS on the three age groups.** Significant differences were assessed by Mann-Whitney U test where “a” refers to changes between elderly and young, “b” refers to changes between centenarians and elderly, “c” between centenarians and young and marked as follows: *p<0.05., **p<0.01, ***p<0.001. Orange color refers to increased concentration, blue color refers to decreased concentration.
(DOCX)

Table S4 **All significantly regulated metabolites in blood serum (mean values ± SD) from the targeted MS on the three age groups from males individual.** Assignment of statistically significant metabolites follow figure legend S3.
(DOCX)

Table S5 **All significantly regulated metabolites in blood serum (mean values ± SD) from the targeted MS on the three age groups from females individual.** Assignment of statistically significant metabolites follow figure legend S3.
(DOCX)

Table S6 **Concentration levels (ng/100 µl serum) of inflammatory markers in serum (mean values ± SD) for the 3 age groups analyzed by UPLC-ESI-MS/MS.** Significant differences were assessed by Mann-Whitney U test where “a” refers to changes in elderly vs young, “b” centenarians vs elderly, “c” centenarians vs young and marked as follows: *p<0.05., **p<0.01, ***p<0.001.Orange color refers to increased concentration, blue color refers to decreased concentration in respect of elderly.
(DOCX)

Table S7 **Concentration levels (ng/100 µl serum) of inflammatory markers in serum (mean values ± SD) for the 3 age groups from females individuals analyzed by UPLC-ESI-MS/MS.** Assignment of statistically significant peaks follow table legend S6.
(DOCX)

Table S8 **Concentration levels (ng/100 µl serum) of inflammatory markers in serum (mean values ± SD) for the 3 age groups from males individuals analyzed by UPLC-ESI-MS/MS.** Assignment of statistically significant peaks follow table legend S6.
(DOCX)

Table S9 **All significantly regulated metabolites in blood serum (mean values ± SD) from the targeted MS on the elderly group.** Significant differences were assessed by Mann-Whitney U test where: *p<0.05, **p<0.01, ***p<0.001. Blue color refers to decreased concentration.
(DOCX)

Table S10 **O-PLS-DA model summary for discriminating urine metabolic profiles.**
(DOCX)

Table S11 **Peak integrals (as a.u = area under) for significantly regulated metabolites in urine for the 3 age groups as detected by ^1^H-NMR profiling.** Assignment of statistically significant peaks was based on δ ^1^H: chemical shifts calibrated against the TSP signal at δ 0.0. s: singlet, d: doublet, t: triplet, m: multiplet. Statistical significance differences are displayed as by Wilcoxon Rank Sum test (***p<0.001 centenarians vs elderly). PAG = phenylacetylglutamine, PCS = p-cresol sulfate, 3-HB = 3-hydroxybenzoate.
(DOCX)

Table S12 **Peak integrals (as a.u = area under) for significantly regulated metabolites in male urines for the 3 age groups as detected by ^1^H-NMR profiling.** Assignment of statistically significant peaks follow figure legend S8.
(DOCX)

Table S13 **Peak integrals (as a.u = area under) for significantly regulated metabolites in female urines for the 3 age groups as detected by ^1^H-NMR profiling.** Assignment of statistically significant peaks follow figure legend S8.
(DOCX)

Table S14 **Correlation coefficients for display markers of aging and longevity and clinical parameters.**
(DOCX)

Table S15 **All significantly regulated metabolites in blood serum (mean values ± SD) from the targeted MS on the Down syndrome individuals.** Significant differences were assessed by Mann-Whitney U test where “a” refers to changes in elderly vs young, “b” centenarians vs elderly, “c” centenarians vs young, “d” Downs vs elderly, “e” Down vs young, “f” Down vs centenarians and marked as follows: *p<0.05., **p<0.01, ***p<0.001. Blue color refers to decreased concentration.
(DOCX)

Text S1 **Use of medications.**
(DOCX)

Text S2 **Hematochemical and cytokines evaluation.**
(DOCX)

Text S3 **Sample preparation for ^1^H NMR Spectroscopy.**
(DOCX)

Text S4 **Validation of biological aging metabolic signatures in Down Syndrome (DS) individuals.**
(DOCX)
