# Correction: Metabolic Signatures of Extreme Longevity in Northern Italian Centenarians Reveal a Complex Remodeling of Lipids, Amino Acids, and Gut Microbiota Metabolism

**DOI:** 10.1371/annotation/5fb9fa6f-4889-4407-8430-6dfc7ecdfbdd

**Published:** 2013-08-13

**Authors:** Sebastiano Collino, Ivan Montoliu, François-Pierre J. Martin, Max Scherer, Daniela Mari, Stefano Salvioli, Laura Bucci, Rita Ostan, Daniela Monti, Elena Biagi, Patrizia Brigidi, Claudio Franceschi, Serge Rezzi

A funding organization and two grants from that organization given to Claudio Franceschi were incorrectly omitted from the Funding Statement. 

The second sentence of the Funding Statement should read: "The research leading to these results has received funding from the European Union's Seventh Framework Programme (FP7/2007-2011) under grant agreement n. 259679 (IDEAL) and KBBE 2010-14 n. 266486 (NU-AGE) to CF, by University of Florence to D. Monti and MIUR (PRIN2006) to D. Monti, CF, and D. Mari." 

