# Supplementary figures and images for: Metabolic Signatures of Extreme Longevity in Northern Italian Centenarians Reveal a Complex Remodeling of Lipids, Amino Acids, and Gut Microbiota Metabolism
Source: PLoS One. 2013 Mar 6;8(3):e56564. doi: 10.1371/journal.pone.0056564 (PMC3590212; doi:10.1371/journal.pone.0056564)

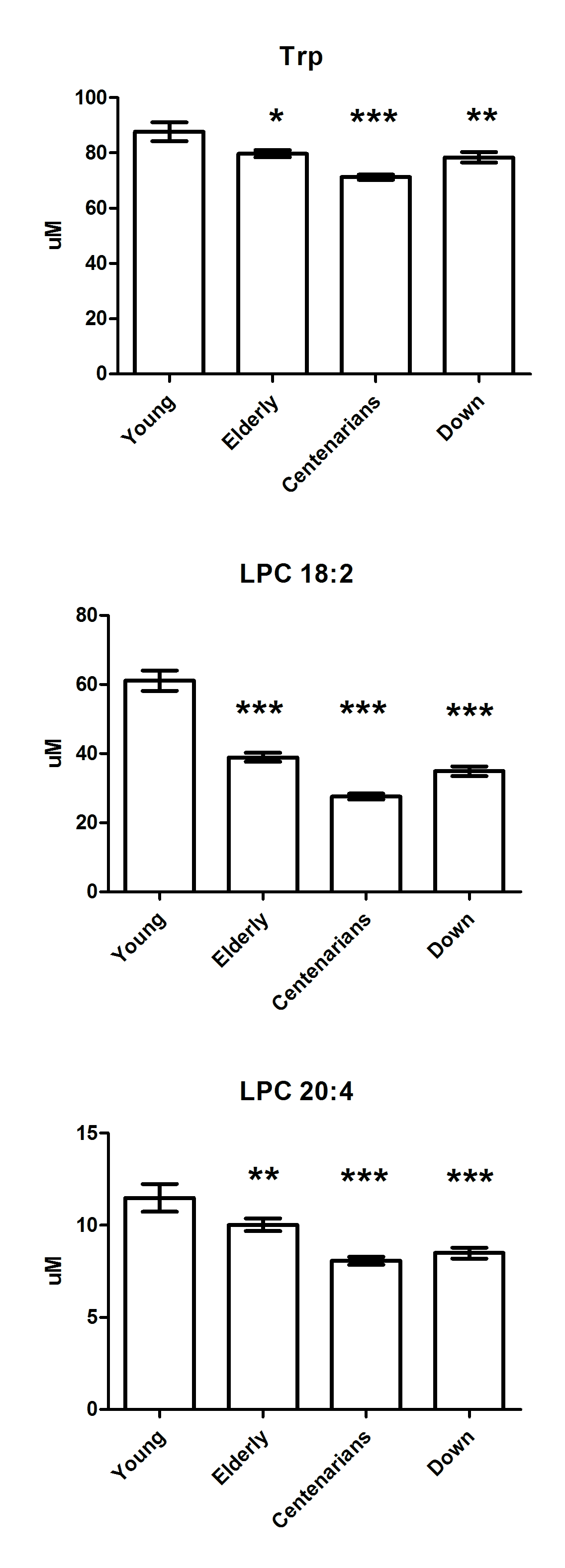

Supplement: Figure S1 — Validation of metabolic signature of biological aging in serum of DS individuals. Bar plots representing mean ±standard error. Despite their young age (mean age 28 yrs), concentration of Trp, LPC 18∶2, and LCP 20∶4 is closer to levels seen in centenarians (LPC 20∶4) and elderly (Trp, LPC 18∶2). All significantly regulated metabolites and statistical changes are listed in Table S4. Significant differences were assessed by Mann-Whitney U test where *p<0.05., **p<0.01, ***p<0.001. (TIF) [file pone.0056564.s001.tif]

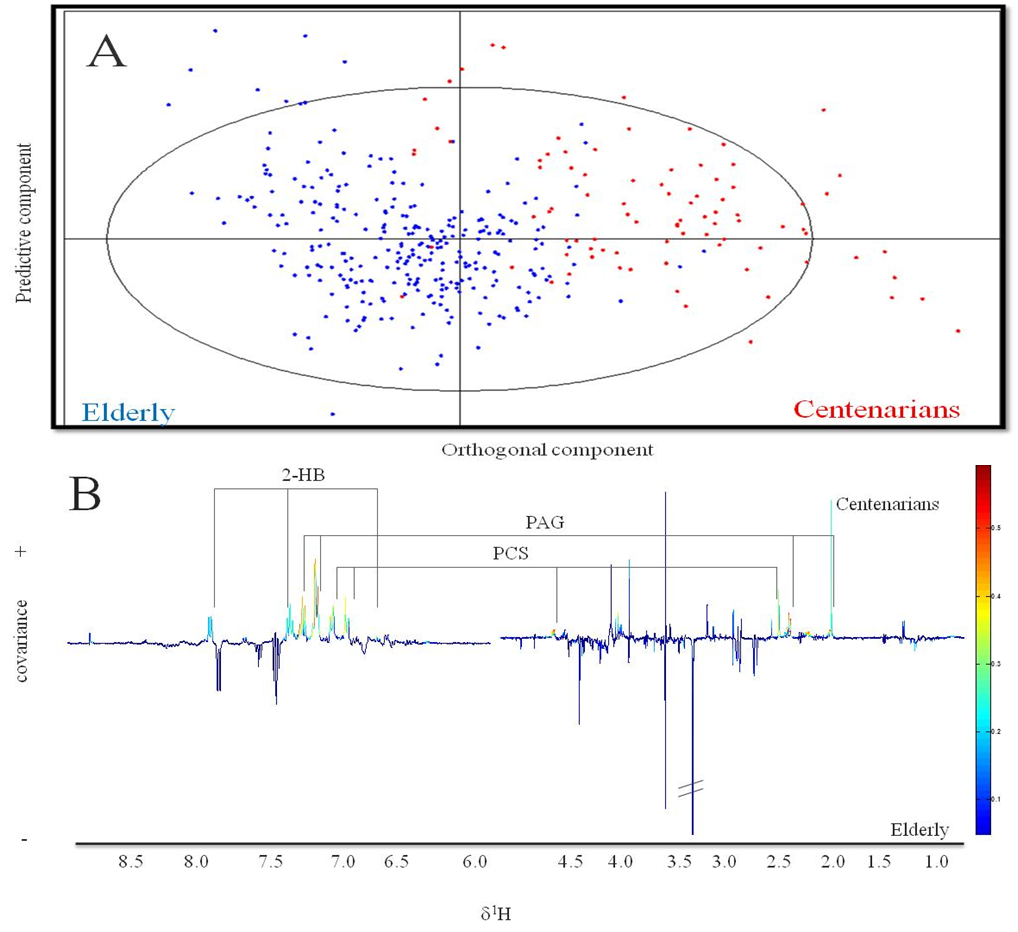

Supplement: Figure S2 — OPLS-DA score (A) and coefficient plots (B) derived from urinary 1H-NMR spectra from elderly (blue) and centenarians (red). PAG = phenylacetylglutamine, PCS = p-cresol sulfate, 3-HB = 3-hydroxybenzoate. (TIFF) [file pone.0056564.s002.tiff]

**Table S14**

**
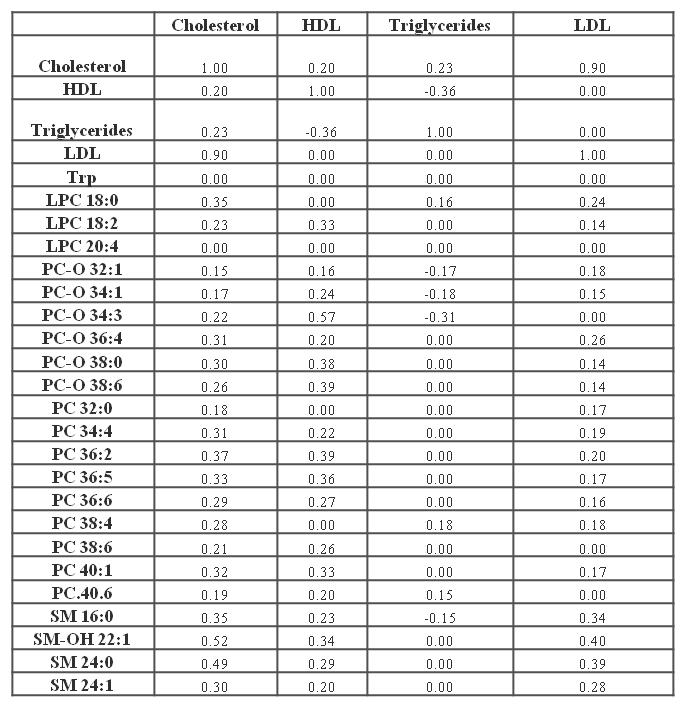
**

Supplement: Table S14 — Correlation coefficients for display markers of aging and longevity and clinical parameters. (DOCX) [file pone.0056564.s016.docx]
