# Supplementary material for: Metabolic Signatures of Extreme Longevity in Northern Italian Centenarians Reveal a Complex Remodeling of Lipids, Amino Acids, and Gut Microbiota Metabolism
Source: PLoS One. 2013 Mar 6;8(3):e56564. doi: 10.1371/journal.pone.0056564 (PMC3590212; doi:10.1371/journal.pone.0056564)
Supplement: Table S2 — Demographic, anthropometric, clinical and haematochemical characteristics of the elderly cohort analyzed by metabolomic targeted MS. Values are presented as mean ±SD with the range in parentheses. Significant differences were assessed by Mann-Whitney U test where no statistical differences are reported between centenarians' offspring and offspring of non long-lived parents. (DOCX) [file pone.0056564.s004.docx]

**Table S2**

| **Factor** | **Elderly-Offspring of centenarians** | **Elderly-Offspring of non long-lived parents** | **Young** |
| --- | --- | --- | --- |
| **Demographic**  Serum-Targeted MS  Gender, *male/female*  Age, *years* | 14/32  68.4^±^6 (56-81) | 19/23  70.7^±^ 6 (59-86) | 11/10  30.9^±^5 (24-40) |
| **Clinical**  BMI, *kg/m^2^*  HOMA  Diabetes^1^, *n*  Cholesterol, *mg/dl*  Triglycerides, *mg/dl*  HDL, *mg/dl*  LDL, *mg/dl*  CRP, mg/L  A-SAA, *μg/ml*  IL-6*, pg/ml*  IL-8*, pg/ml*  IL-10, *pg/ml*  TNF-alpha, *pg/ml*  Leptin, *ng/ml*  Resistin, *ng/ml*  Adiponectin, *μg/ml*  Hypertension, *n*  Cardiovascular therapy, *n*  MMSE^2^  Heart failure, *n*  Irregular heart rhythm, *n*  Angina pectoris, *n* | 26.5^±^4.0 (19.6-39.7)  2.65^±^2.2 (0.5-11)  4  208.0^±^30.2 (154-276)  120.0^±^57.6 (41-550)  52.6^+^16.2 (20-99)  129.1^±^36.1 (101-192)  2.4^±^2.8 (0.29-15.9)  180^±^201.6 (0.01-872)  22.7^±^17.7 (3.5-78.7)  9.05^±^3.93 (3.8-12.9)  4.36^±^5.08 (0.5-20.1)  25.6^±^32.1 (0.1-146)  25.2^±^23.0 (2.01-99.2)  9.84^±^4.4 (4.49-23.4)  42.4^±^35.7 (1.97-155)  15  5  27.5^±^1.3 (24-30)  0  2  2 | 27.8^±^4.6 (25.9-39.1)  2.7^±^1.3 (0.5-6.10)  6  198.0^±^34.1 (124-300)  120.1^±^58.1 (52-332)  58.4^+^20.3 (31-109)  116^±^40.8 (101-127)  3.38^±^4.05 (0.21-20.2)  125.1^±^154 (0.01-937)  39.4^±^62.4 (2.3-286)  16.4^±^17.7 (6.8-29)  4.17^±^4.83 (0.4-23)  60.1^±^5.08 (0.1-690)  26.2^±^27.3 (0.39-94.6)  10.3^±^4.8 (1.6-27.2)  43.1^±^40.7 (0.49-188.3)  25  19  26.8^±^3.1 (9.7-30.0)  1  9  1 | 22.1^±^2.0 (18.3.24.6)  n/a  n/a  162.3^±^28.4 (133-207)  71.7^±^32.1 (28-143)  51.8^±^8.7 (38-66)  89.8^±^51.5 (49-144)  0.72^±^0.4 (0.28-2.08)  n/a  20.3^±^17.5 (2.70-28.2)  19.3^±^13.3 (4.4-46.6)  2.38^±^2.58 (0.80-3.80)  18.5^±^28.5(5.80-65.5)  n/a  n/a  n/a  n/a  n/a  n/a  n/a  n/a  n/a |

Legend: BMI=body mass index, HOMA=Homeostatic Model Assessment index, HDL= high density lipoprotein, LDL= low density lipoprotein,CRP=C reactive protein, A-SAA= Serum amyloid A (SAA) proteins,IL-6= Interleukin-6, IL-10= Interleukin-10, TNF-alpha= Tumor necrosis factor.

^1^Diabetes mellitus: history of diabetes, fasting glucose plasma ≥126mg/dl.

^2^MMSE= Cognitive function measure using the Mini-Mental State Examination (MMSE). The score used in the analysis was corrected by age and years of educations according to Magni et. al for old people.MMSE for elderly cognitive impairment was graded as severe (score 0–17), mild (score 18–23), or not present (score 24–30). MMSE for centenarians ≥ 20 absence of severe cognitive decline; <12 presence of severe cognitive decline according to Franceschi et al.2000a.
