# Supplementary material for: Metabolic Signatures of Extreme Longevity in Northern Italian Centenarians Reveal a Complex Remodeling of Lipids, Amino Acids, and Gut Microbiota Metabolism
Source: PLoS One. 2013 Mar 6;8(3):e56564. doi: 10.1371/journal.pone.0056564 (PMC3590212; doi:10.1371/journal.pone.0056564)
Supplement: Table S3 — All significantly regulated metabolites in blood serum (mean values ± SD) from the targeted MS on the three age groups. Significant differences were assessed by Mann-Whitney U test where “a” refers to changes between elderly and young, “b” refers to changes between centenarians and elderly, “c” between centenarians and young and marked as follows: *p<0.05., **p<0.01, ***p<0.001. Orange color refers to increased concentration, blue color refers to decreased concentration. (DOCX) [file pone.0056564.s005.docx]

**Table S3**

| Metabolites [μM/l] | Young | Elderly | Centenarians |
| --- | --- | --- | --- |
|  | Mean ± SD | Mean ± SD | Mean ± SD |
| Trp | 87.7 ± 15.2 | 80.5 ± 12.7 ^a(*)^ | 71.3 ± 11.5^b(***),c(***)^ |
| PC32:0 | 9.58 ± 1.68 | 11.2 ± 2.39 ^a(**)^ | 12.7 ± 2.39 ^b(***),c(***)^ |
| PC34:4 | 0.86 ± 0.29 | 1.21 ± 0.45 ^a(***)^ | 0.85 ± 0.34 ^b(***)^ |
| PC36:5 | 9.39 ± 2.44 | 18.2 ± 15.4 ^a(***)^ | 11.7 ± 5.33 ^b(***)^ |
| PC36:6 | 0.38 ± 0.13 | 0.60 ± 0.31 ^a(**)^ | 0.40 ± 0.18 ^b(***)^ |
| PC38:4 | 78.4 ± 14.1 | 103 ± 28.3 ^a(***)^ | 85.3 ± 22.8 ^b(***)^ |
| PC38:6 | 46.4 ± 10.3 | 64.7 ± 22.9 ^a(***)^ | 52.8 ± 22.2 ^b(***)^ |
| PC40:6 | 15.0 ± 4.51 | 23.7 ± 8.95 ^a(***)^ | 18.9 ± 8.28 ^b(***)^ |
| PC-O 32:1 | 2.02 ± 0.36 | 2 ± 0.51 | 2.35 ± 0.63 ^b(***),c(***)^ |
| PC-O 34:1 | 7.34 ± 1.07 | 7.88 ± 1.71 | 9.54 ± 2.19 ^b(***),c(***)^ |
| PC-O 34:3 | 5.73 ± 1.4 | 5.07 ± 1.71 | 3.94 ± 1.54 ^b(***),c(***)^ |
| PC-O 36:2 | 9.54 ± 1.75 | 9.58 ± 2.39 | 9.29 ± 2.26 ^c (*)^ |
| PC-O 36:4 | 14.5 ± 2.83 | 14.4 ± 3.55 | 12.4 ± 2.56 ^b(***),c(***)^ |
| PC-O 38.0 | 1.27 ± 0.29 | 1.78 ± 0.64 ^a(***)^ | 1.31 ± 0.51^b(***)^ |
| PC-O 38:6 | 4.64 ± 1.07 | 5.61 ± 1.51 ^a(***)^ | 4.61 ± 1.37^b(***)^ |
| PC-O 40:1 | 1.23 ± 0.23 | 1.41 ± 0.41 | 1.02 ± 0.32^b(***),c(***)^ |
| LPC 18:0 | 52.2 ± 12.9 | 52.1 ± 13.5 | 40.4 ± 12.0^b(***),c(***)^ |
| LPC 18:2 | 61.1 ± 13.1 | 39.04 ± 12.4 ^a(***)^ | 27.6 ± 10.3^b(***),c***)^ |
| LPC 20:4 | 11.5 ± 3.38 | 10.1 ± 3.23 ^a(*)^ | 8.07 ± 2.51^b(***),c(***)^ |
| SM 16:0 | 119 ± 17.6 | 127.0 ± 22.6 ^a(*)^ | 138 ± 24.1^b(**),c(***)^ |
| SM 24:0 | 23.4 ± 4.37 | 25.6 ± 5.31 | 19.8 ± 4.92^b(***),c(**)^ |
| SM 24:1 | 55.9 ± 6.81 | 65.7 ± 10.9 ^a(***)^ | 69.8 ± 12.6^b(*),c(***)^ |
| SM-OH 22:1 | 14.5 ± 2.94 | 16.1 ± 3.37 | 11.5 ± 3.04^b(***),c(***)^ |
