# Supplementary material for: Metabolic Signatures of Extreme Longevity in Northern Italian Centenarians Reveal a Complex Remodeling of Lipids, Amino Acids, and Gut Microbiota Metabolism
Source: PLoS One. 2013 Mar 6;8(3):e56564. doi: 10.1371/journal.pone.0056564 (PMC3590212; doi:10.1371/journal.pone.0056564)
Supplement: Table S4 — All significantly regulated metabolites in blood serum (mean values ± SD) from the targeted MS on the three age groups from males individual. Assignment of statistically significant metabolites follow figure legend S3. (DOCX) [file pone.0056564.s006.docx]

**Table S4**

| Metabolites [μM/l] | Young-Males | Elderly-Males | Centenarians-Males |
| --- | --- | --- | --- |
|  | Mean ± SD | Mean ± SD | Mean ± SD |
| Trp | 90.9 ± 14.1 | 81.7 ± 13.9 ^a(*)^ | 74.9 ± 10.2 ^b (*),c(**)^ |
| PC32:0 | 9.49 ± 2.16 | 10.9 ± 1.91 ^a(**)^ | 12.4 ± 2.15 ^b(**),c(***)^ |
| PC34:4 | 0.90 ± 0.32 | 1.11 ± 0.36 ^a(***)^ | 0.77 ± 0.24 ^b(***)^ |
| PC36:5 | 9.91 ± 2.73 | 22.9 ± 23.1 ^a(***)^ | 11.7 ± 5.41 ^b(***)^ |
| PC36:6 | 0.41 ± 0.15 | 0.65 ± 0.33 ^a(**)^ | 0.35 ± 0.17 ^b(***)^ |
| PC38:4 | 81.6 ± 14.3 | 93.9 ± 23.8 ^a(***)^ | 82.1 ± 20.7 ^b(*)^ |
| PC38:6 | 46.8 ± 8.83 | 68.5 ± 24.6 ^a(***)^ | 55.0 ± 25.4 ^b(***)^ |
| PC40:6 | 14.6 ± 2.28 | 23.6 ± 9.77 ^a(***)^ | 19.1 ± 8.46 ^b(***)^ |
| PC-O 32:1 | 1.96 ± 0.38 | 1.91 ± 0.41 | 2.23 ± 0.44 ^b(***),c(***)^ |
| PC-O 34:1 | 6.94 ± 0.87 | 7.53 ± 1.48 | 9.25 ± 1.84 ^b(***),c(***)^ |
| PC-O 34:3 | 5.62 ± 1.52 | 4.65 ± 1.33 | 4.04 ± 1.17 ^c(***)^ |
| PC-O 36:2 | 9.02 ± 1.55 | 8.83 ± 1.71 | 9.05 ±1.72 |
| PC-O 36:4 | 14.6 ± 3.82 | 13.8 ± 2.94 | 12.3 ± 2.01 ^b(***),c(***)^ |
| PC-O 38.0 | 1.28 ± 0.31 | 1.89 ± 0.71 ^a(***)^ | 1.31 ± 0.45^b(***)^ |
| PC-O 38:6 | 4.69 ± 1.06 | 5.71 ± 1.63 ^a(***)^ | 4.62 ± 1.27^b(***)^ |
| PC-O 40:1 | 1.43 ± 0.41 | 1.36 ± 0.36 | 1.05 ± 0.26^b(***),c(***)^ |
| LPC 18:0 | 53.6 ± 13.5 | 49.1 ± 12.9 | 41.9 ± 10.49^b(*),c(***)^ |
| LPC 18:2 | 65.2 ± 11.0 | 40.9 ± 12.1 ^a(***)^ | 31.9 ± 11.3^b(***),c***)^ |
| LPC 20:4 | 13.12 ± 2.86 | 10.4 ± 2.88 ^a(**)^ | 7.87 ± 2.47^b(***),c(***)^ |
| SM 16:0 | 115 ± 13.9 | 124 ± 19.7 ^a(*)^ | 136 ± 23.1^b(**),c(***)^ |
| SM 24:0 | 24.5 ± 4.90 | 25.9 ± 4.90 | 20.0 ± 5.59^b(***),c(**)^ |
| SM 24:1 | 56.1 ± 7.16 | 66.4 ± 9.04 ^a(***)^ | 71.1 ± 13.9^b(*),c(***)^ |
| SM-OH 22:1 | 14.5 ± 2.95 | 15.7 ± 2.85 | 11.1 ± 3.09^b(***),c(***)^ |
