# Supplementary material for: Metabolic Signatures of Extreme Longevity in Northern Italian Centenarians Reveal a Complex Remodeling of Lipids, Amino Acids, and Gut Microbiota Metabolism
Source: PLoS One. 2013 Mar 6;8(3):e56564. doi: 10.1371/journal.pone.0056564 (PMC3590212; doi:10.1371/journal.pone.0056564)
Supplement: Table S5 — All significantly regulated metabolites in blood serum (mean values ± SD) from the targeted MS on the three age groups from females individual. Assignment of statistically significant metabolites follow figure legend S3. (DOCX) [file pone.0056564.s007.docx]

**Table S5**

| Metabolites [μM/l] | Young-Females | Elderly-Females | Centenarians-Females |
| --- | --- | --- | --- |
|  | Mean ± SD | Mean ± SD | Mean ± SD |
| Trp | 84.0 ± 16.5 | 79.7 ± 11.9 ^a(**)^ | 70.3 ± 11.6^b(***),c(***)^ |
| PC32:0 | 9.67 ± 0.92 | 11.3 ± 2.62 ^a(**)^ | 12.8 ± 2.45 ^b(***),c(***)^ |
| PC34:4 | 0.81 ± 0.25 | 1.28 ± 0.48 ^a(***)^ | 0.86 ± 0.36 ^b(***)^ |
| PC36:5 | 8.64 ± 1.94 | 15.3 ± 6.96 ^a(***)^ | 11.7 ± 5.32 ^b(***)^ |
| PC36:6 | 0.35 ± 0.08 | 0.57 ± 0.26 ^a(**)^ | 0.41 ± 0.18 ^b(***)^ |
| PC38:4 | 74.5 ± 13.5 | 109 ± 29.6 ^a(***)^ | 87.6 ± 23.3 ^b(***)^ |
| PC38:6 | 45.9 ± 12.43 | 62.7 ± 21.7 ^a(***)^ | 52.2 ± 21.3 ^b(***)^ |
| PC40:6 | 15.5 ± 6.41 | 23.8 ± 8.48 ^a(***)^ | 18.9 ± 8.27 ^b(***)^ |
| PC-O 32:1 | 2.09 ± 0.35 | 2.06 ± 0.55 | 2.37 ± 0.66 ^b(***),c(***)^ |
| PC-O 34:1 | 7.82 ± 1.13 | 8.07 ± 1.81 | 9.61 ± 2.27 ^b(***),c(***)^ |
| PC-O 34:3 | 5.86 ± 1.31 | 5.31 ± 1.84 | 3.90 ± 1.62 ^b(***),c(***)^ |
| PC-O 36:2 | 10.1 ± 1.67 | 10.04 ± 2.61 | 9.35 ± 2.41 ^c(*)^ |
| PC-O 36:4 | 13.6 ± 1.73 | 14.6 ± 3.82 | 12.4 ± 2.68 ^b(***),c(***)^ |
| PC-O 38.0 | 1.24 ± 0.21 | 1.43 ± 0.41 ^a(***)^ | 1.37 ± 0.51^b(***)^ |
| PC-O 38:6 | 4.58 ± 1.13 | 5.53 ± 1.43 ^a(***)^ | 4.59 ± 1.36^b(***)^ |
| PC-O 40:1 | 1.23 ± 0.22 | 1.43 ± 0.41 | 1.02 ± 0.32^b(***),c(***)^ |
| LPC 18:0 | 50.4 ± 16.1 | 53.7 ± 13.5 | 39.9 ± 12.24^b(***),c(***)^ |
| LPC 18:2 | 56.1 ± 11.7 | 37.7 ± 12.5 ^a(***)^ | 26.4 ± 9.77^b(***),c***)^ |
| LPC 20:4 | 9.48 ± 2.99 | 9.82 ± 3.41 | 7.87 ± 2.47^b(***),c(***)^ |
| SM 16:0 | 124 ± 21.1 | 129 ± 24.1 ^a(*)^ | 139 ± 24.4^b(**),c(***)^ |
| SM 24:0 | 22.1 ± 3.45 | 25.1 ± 5.91 | 19.7 ± 4.89^b(***),c(**)^ |
| SM 24:1 | 55.8 ± 6.76 | 65.8 ± 12.3 ^a(***)^ | 69.6 ± 12.26^b(*),c(***)^ |
| SM-OH 22:1 | 14.5 ± 3.10 | 16.4 ± 3.80 | 11.6 ± 3.02^b(***),c(***)^ |
