# Supplementary material for: Metabolic Signatures of Extreme Longevity in Northern Italian Centenarians Reveal a Complex Remodeling of Lipids, Amino Acids, and Gut Microbiota Metabolism
Source: PLoS One. 2013 Mar 6;8(3):e56564. doi: 10.1371/journal.pone.0056564 (PMC3590212; doi:10.1371/journal.pone.0056564)
Supplement: Table S6 — Concentration levels (ng/100 µl serum) of inflammatory markers in serum (mean values ± SD) for the 3 age groups analyzed by UPLC-ESI-MS/MS. Significant differences were assessed by Mann-Whitney U test where “a” refers to changes in elderly vs young, “b” centenarians vs elderly, “c” centenarians vs young and marked as follows: *p<0.05., **p<0.01, ***p<0.001.Orange color refers to increased concentration, blue color refers to decreased concentration in respect of elderly. (DOCX) [file pone.0056564.s008.docx]

**Table S6**

| Metabolites [(ng/100 μl serum] | Young | Elderly | Centenarians |
| --- | --- | --- | --- |
| LTE4 | 0.015 ± 0.014 | 0.013 ± 0.011 | 0.035 ± 0.031 ^b(*),c(*)^ |
| EPA | 0.097 ± 0.036 | 0.123 ± 0.052 | 0.078 ± 0.026 ^b(***)^ |
| 15-HETE | 1.512 ± 1.949 | 1.255 ± 1.245 | 3.348 ± 2.865 ^b(*),c(*)^ |
| 11,12-DiHETrE | 0.017 ± 0.006 | 0.017 ± 0.004 | 0.015 ± 0.006 ^c(*)^ |
| 9-oxo-ODE | 0.042 ± 0.028 | 0.043 ± 0.039 | 0.022 ± 0.013 ^b(***),c(***)^ |
| 9-HODE | 0.348 ± 0.223 | 0.397 ± 0.677 | 0.204 ± 0.211 ^b(*)^ |
| 8,9-EpETrE | 0.067 ± 0.101 | 0.074 ± 0.186 | 0.113 ± 0.107 ^b(*)^ |
