# Supplementary material for: Metabolic Signatures of Extreme Longevity in Northern Italian Centenarians Reveal a Complex Remodeling of Lipids, Amino Acids, and Gut Microbiota Metabolism
Source: PLoS One. 2013 Mar 6;8(3):e56564. doi: 10.1371/journal.pone.0056564 (PMC3590212; doi:10.1371/journal.pone.0056564)
Supplement: Table S7 — Concentration levels (ng/100 µl serum) of inflammatory markers in serum (mean values ± SD) for the 3 age groups from females individuals analyzed by UPLC-ESI-MS/MS. Assignment of statistically significant peaks follow table legend S6. (DOCX) [file pone.0056564.s009.docx]

**Table S7**

| Metabolites [(ng/100 μl serum] | Young-Females | Elderly-Females | Centenarians-Females |
| --- | --- | --- | --- |
| LTE4 | 0.013 ± 0.015 | 0.014 ± 0.011 | 0.038 ± 0.031 ^b(*),c(*)^ |
| EPA | 0.082 ± 0.043 | 0.129 ± 0.046 | 0.080 ± 0.026 ^b(**)^ |
| 15-HETE | 1.992 ± 2.645 | 1.371 ± 1.621 | 3.168 ± 3.133 ^b(*),c(*)^ |
| 11,12-DiHETrE | 0.019 ± 0.006 | 0.016 ± 0.003 | 0.014 ± 0.006 ^c(*)^ |
| 9-oxo-ODE | 0.030 ± 0.013 | 0.052 ± 0.051 | 0.023 ± 0.014 ^b(***)^ |
| 9-HODE | 0.296 ± 0.221 | 0.431 ± 0.543 | 0.217± 0.229 ^b(*)^ |
| 8,9-EpETrE | 0.051 ± 0.035 | 0.054 ± 0.053 | 0.116 ± 0.117 ^b(*)^ |
