# Supplementary material for: Metabolic Signatures of Extreme Longevity in Northern Italian Centenarians Reveal a Complex Remodeling of Lipids, Amino Acids, and Gut Microbiota Metabolism
Source: PLoS One. 2013 Mar 6;8(3):e56564. doi: 10.1371/journal.pone.0056564 (PMC3590212; doi:10.1371/journal.pone.0056564)
Supplement: Table S8 — Concentration levels (ng/100 µl serum) of inflammatory markers in serum (mean values ± SD) for the 3 age groups from males individuals analyzed by UPLC-ESI-MS/MS. Assignment of statistically significant peaks follow table legend S6. (DOCX) [file pone.0056564.s010.docx]

**Table S8**

| Metabolites [(ng/100 μl serum] | Young-Males | Elderly-Males | Centenarians-Males |
| --- | --- | --- | --- |
| LTE4 | 0.017 ± 0.001 | 0.012 ± 0.011 | 0.017 ± 0.031 |
| EPA | 0.113 ± 0.020 | 0.118 ± 0.056 | 0.068 ± 0.022 ^b(*)^ |
| 15-HETE | 1.033 ± 0.734 | 1.167 ± 0.893 | 4.255 ± 0.131 ^b(^^**),c(**)^ |
| 11,12-DiHETrE | 0.020 ± 0.006 | 0.018 ± 0.004 | 0.015 ± 0.005 ^c(*)^ |
| 9-oxo-ODE | 0.054 ± 0.035 | 0.035 ± 0.025 | 0.019 ± 0.002 ^b(*)^ |
| 9-HODE | 0.400 ± 0.223 | 0.371 ± 0.777 | 0.143 ± 0.008 ^b(*)^ |
| 8,9-EpETrE | 0.083 ± 0.141 | 0.090 ± 0.243 | 0.097 ± 0.024 |
