# Supplementary material for: Metabolic Signatures of Extreme Longevity in Northern Italian Centenarians Reveal a Complex Remodeling of Lipids, Amino Acids, and Gut Microbiota Metabolism
Source: PLoS One. 2013 Mar 6;8(3):e56564. doi: 10.1371/journal.pone.0056564 (PMC3590212; doi:10.1371/journal.pone.0056564)
Supplement: Table S9 — All significantly regulated metabolites in blood serum (mean values ± SD) from the targeted MS on the elderly group. Significant differences were assessed by Mann-Whitney U test where: *p<0.05, **p<0.01, ***p<0.001. Blue color refers to decreased concentration. (DOCX) [file pone.0056564.s011.docx]

**Table S9**

| Metabolites [μM/l] | Elderly-Offspring of centenarians | Elderly-Offspring of non long-lived parents |
| --- | --- | --- |
|  | Mean ± SD | Mean ± SD |
| LPC 16:0 | 175.9 ± 36.9 | 157.9 ± 38.4^**^ |
| LPC 16:1 | 4.66 ± 1.32 | 4.02 ± 1.67^**^ |
| LPC 18:0 | 56.3 ± 13.7 | 47.6 ± 11.8^**^ |
| LPC 18:1 | 39.1 ± 10.4 | 33.5 ± 9.55^**^ |
| LPC 18:2 | 42.5 ± 12.2 | 35.11 ± 11.6^**^ |
| Ser | 133.3 ± 23.0 | 121.8 ± 25.1^**^ |
| Phe | 68.2 ± 11.9 | 63.1 ± 11.3^*^ |
| PC-O 36:3 | 5.24 ± 1.35 | 4.77 ± 1.08^*^ |
