# Supplementary material for: Metabolic Signatures of Extreme Longevity in Northern Italian Centenarians Reveal a Complex Remodeling of Lipids, Amino Acids, and Gut Microbiota Metabolism
Source: PLoS One. 2013 Mar 6;8(3):e56564. doi: 10.1371/journal.pone.0056564 (PMC3590212; doi:10.1371/journal.pone.0056564)
Supplement: Table S10 — O-PLS-DA model summary for discriminating urine metabolic profiles. (DOCX) [file pone.0056564.s012.docx]

**Table S10**.

| **Overview** | **R2X_(cum)_** | **R2Y_(cum)_** | **Q^2^Y** | **AuROC** | |
| --- | --- | --- | --- | --- | --- |
| Centenarians vs. Elderly | 0.14 | 0.52 | 0.39 | 0.96 | 0.93 |
| Centenarians vs. Young | 0.14 | 0.86 | 0.75 | 1.00 | 1.00 |
| Young vs. Elderly | 0.05 | 0.21 | 0.09 | 0.92 | 0.81 |
