# Supplementary material for: Metabolic Signatures of Extreme Longevity in Northern Italian Centenarians Reveal a Complex Remodeling of Lipids, Amino Acids, and Gut Microbiota Metabolism
Source: PLoS One. 2013 Mar 6;8(3):e56564. doi: 10.1371/journal.pone.0056564 (PMC3590212; doi:10.1371/journal.pone.0056564)
Supplement: Table S11 — Peak integrals (as a.u = area under) for significantly regulated metabolites in urine for the 3 age groups as detected by 1H-NMR profiling. Assignment of statistically significant peaks was based on δ 1H: chemical shifts calibrated against the TSP signal at δ 0.0. s: singlet, d: doublet, t: triplet, m: multiplet. Statistical significance differences are displayed as by Wilcoxon Rank Sum test (***p<0.001 centenarians vs elderly). PAG = phenylacetylglutamine, PCS = p-cresol sulfate, 3-HB = 3-hydroxybenzoate. (DOCX) [file pone.0056564.s013.docx]

**Table S11**

|  |  | **Centenarians** | **Elderly** | **Young** |
| --- | --- | --- | --- | --- |
| **Peak Integral (a.u.)** | **Chemical shift** | **Mean ± SD** | **Mean ± SD** | **Mean ± SD** |
| **PAG** | 7.43 (m), 7.36 (m)  4.18 (m), 2.27 (t),  2.11 (m) | 9.93 ± 3.72*** | 6.62 ± 2.59 | 5.89 ± 2.35 |
| **PCS** | 2.34 (s), 7.21 (d),  7.28 (d)) | 4.06 ± 1.53*** | 2.62 ± 1.22 | 2.32 ± 0.85 |
| **2-HB** | 7.87 (d), 7.49 (m), 7.02, 6.95 (m) | 1.60 ± 2.16*** | 0.51 ± 0.92 | 0.46 ± 0.05 |
