# Supplementary material for: Metabolic Signatures of Extreme Longevity in Northern Italian Centenarians Reveal a Complex Remodeling of Lipids, Amino Acids, and Gut Microbiota Metabolism
Source: PLoS One. 2013 Mar 6;8(3):e56564. doi: 10.1371/journal.pone.0056564 (PMC3590212; doi:10.1371/journal.pone.0056564)
Supplement: Table S12 — Peak integrals (as a.u = area under) for significantly regulated metabolites in male urines for the 3 age groups as detected by 1H-NMR profiling. Assignment of statistically significant peaks follow figure legend S8. (DOCX) [file pone.0056564.s014.docx]

**Table S12**

|  |  | **Centenarians Males** | **Elderly**  **Males** | **Young**  **Males** |
| --- | --- | --- | --- | --- |
| **Peak Integral (a.u.)** | **Chemical shift** | **Mean ± SD** | **Mean ± SD** | **Mean ± SD** |
| **PAG** | 7.43 (m), 7.36 (m)  4.18 (m), 2.27 (t),  2.11 (m) | 9.56 ± 3.42*** | 6.15 ± 2.38 | 6.47 ± 2.25 |
| **PCS** | 2.34 (s), 7.21 (d),  7.28 (d)) | 3.89 ± 1.49*** | 2.43 ± 1.01 | 2.55 ± 0.85 |
| **2-HB** | 7.87 (d), 7.49 (m), 7.02, 6.95 (m) | 14.7 ± 2.84*** | 2.43 ± 1.06 | 0.32 ± 0.81 |
