# Supplementary material for: Metabolic Signatures of Extreme Longevity in Northern Italian Centenarians Reveal a Complex Remodeling of Lipids, Amino Acids, and Gut Microbiota Metabolism
Source: PLoS One. 2013 Mar 6;8(3):e56564. doi: 10.1371/journal.pone.0056564 (PMC3590212; doi:10.1371/journal.pone.0056564)
Supplement: Table S13 — Peak integrals (as a.u = area under) for significantly regulated metabolites in female urines for the 3 age groups as detected by 1H-NMR profiling. Assignment of statistically significant peaks follow figure legend S8. (DOCX) [file pone.0056564.s015.docx]

**Table S13**

|  |  | **Centenarians Females** | **Elderly**  **Females** | **Young**  **Females** |
| --- | --- | --- | --- | --- |
| **Peak Integral (a.u.)** | **Chemical shift** | **Mean ± SD** | **Mean ± SD** | **Mean ± SD** |
| **PAG** | 7.43 (m), 7.36 (m)  4.18 (m), 2.27 (t),  2.11 (m) | 10.1 ± 3.79*** | 7.01 ± 2.69 | 4.62 ± 1.24 |
| **PCS** | 2.34 (s), 7.21 (d),  7.28 (d)) | 4.11 ± 1.55*** | 2.78 ± 1.37 | 2.02 ± 0.75 |
| **2-HB** | 7.87 (d), 7.49 (m), 7.02, 6.95 (m) | 1.63 ± 1.98*** | 0.49 ± 3.88 | 0.65 ± 0.07 |
