# Supplementary material for: Metabolic Signatures of Extreme Longevity in Northern Italian Centenarians Reveal a Complex Remodeling of Lipids, Amino Acids, and Gut Microbiota Metabolism
Source: PLoS One. 2013 Mar 6;8(3):e56564. doi: 10.1371/journal.pone.0056564 (PMC3590212; doi:10.1371/journal.pone.0056564)
Supplement: Table S15 — All significantly regulated metabolites in blood serum (mean values ± SD) from the targeted MS on the Down syndrome individuals. Significant differences were assessed by Mann-Whitney U test where “a” refers to changes in elderly vs young, “b” centenarians vs elderly, “c” centenarians vs young, “d” Downs vs elderly, “e” Down vs young, “f” Down vs centenarians and marked as follows: *p<0.05., **p<0.01, ***p<0.001. Blue color refers to decreased concentration. (DOCX) [file pone.0056564.s017.docx]

**Table S15**

| Metabolites [μM/l] | Young | Elderly | Centenarians | Down |
| --- | --- | --- | --- | --- |
|  | Mean ± SD | Mean ± SD | Mean ± SD | Mean ± SD |
| Trp | 87.7 ± 15.2 | 80.5 ± 12.7 ^a(*)^ | 71.3 ± 11.5^b(***),c(***)^ | 78.4 ± 13.5 ^e(**),f(**)^ |
| LPC 18:2 | 61.1 ± 13.05 | 39.0 ± 12.4 ^a(***)^ | 27.6 ± 10.3^b(***),c***)^ | 34.9 ± 10.1 ^e(***),f(***)^ |
| LPC 20:4 | 11.5 ± 3.38 | 10.0 ± 3.23 ^a(*)^ | 8.07 ± 2.51^b(***),c(***)^ | 8.48± 2.2 ^d(***),e(***)^ |
| PC32:0 | 9.58 ± 1.68 | 11.2 ± 2.39 ^a(**)^ | 12.7 ± 2.39 ^b(***),c(***)^ | 9.32± 2.26 ^d(***),f(***)^ |
| SM 16:0 | 119.1 ± 17.6 | 127.0 ± 22.6 ^a(*)^ | 138.3 ± 24.1^b(**),c(***)^ | 109.7±21.3 ^d(***),f(***)^ |
| SM 24:1 | 55.9 ± 6.81 | 65.7 ± 10.9 ^a(***)^ | 69.9 ± 12.6^b(*),c(***)^ | 55.2±11.8 ^d(***),f(***)^ |
