# Supplementary material for: Metabolic Signatures of Extreme Longevity in Northern Italian Centenarians Reveal a Complex Remodeling of Lipids, Amino Acids, and Gut Microbiota Metabolism
Source: PLoS One. 2013 Mar 6;8(3):e56564. doi: 10.1371/journal.pone.0056564 (PMC3590212; doi:10.1371/journal.pone.0056564)
Supplement: Text S1 — Use of medications. (DOCX) [file pone.0056564.s018.docx]

**Text S1**

Current use of medications (including inspection of the drugs by the interviewer) was recorded and drugs grouped in 4 main therapies: cardiovascular (anti-arrhythmic and/or vasodilatator and/or thrombolytic drugs), hypotensive (calcium channel blockers and/or ACE inhibitors and/or diuretics and/or beta blockers), antidiabetic (sulfonylureas and/or biguanides and/or insulin and insulin analogues and/or other oral antidiabetic association), and lipid-lowering (statins and/or fibrates and/or other hypolipidemic agents) therapy. Subjects affected by malignant neoplasia and/or in therapy with immunosuppressor drugs (like cyclosporine, methotrexate, glucocorticoid, etc.) or anti-coagulant drugs were excluded from the study. As far as DS subjects, further exclusion criteria were current acute illnesses, hepatic, renal and cardiac insufficiency, assumption of antioxidant or nutraceutical substances (vitamins, lipoic acid, acetylcysteine, omega 3 and 6 fatty acids, probiotics) within the last two months.
