# Supplementary material for: Metabolic Signatures of Extreme Longevity in Northern Italian Centenarians Reveal a Complex Remodeling of Lipids, Amino Acids, and Gut Microbiota Metabolism
Source: PLoS One. 2013 Mar 6;8(3):e56564. doi: 10.1371/journal.pone.0056564 (PMC3590212; doi:10.1371/journal.pone.0056564)
Supplement: Text S2 — Hematochemical and cytokines evaluation. (DOCX) [file pone.0056564.s019.docx]

**Text S2**

Overnight fasting blood samples were obtained early in the morning Total serum cholesterol, HDL, triglycerides, and CRP were determined using routine laboratory methods. The concentration of serum LDL was calculated by using the Friedewald equation: LDL = total cholesterol – HDL – (triglycerides/). Insulin resistance status was assessed as homeostasis model assessment of insulin resistance (HOMA-IR) according to the previously described formula: insulin (μU/mL) x glucose (mmol/L) / 22.5. Plasma levels of IL-6, IL-10, TNF-α trimer, IL-8, and serum amyloid A were measured by multiplex sandwich ELISA technology (SearchLight, Aushon Biosystems, Billerica, MA) according to the manufacturer’s instructions. Samples, standards, and reagents were dispensed in the plates by a standardized technique employing a robotic liquid handling system with 16 channels (Microlab® STAR, Hamilton Robotics, Reno, NV). Plasma TGF-β1 concentration was determined by ELISA using a commercial kit (DRG Instruments GmbH, Marburg, Germany) according to the manufacturer’s instructions. Concentration of TGF-β1 was detected and quantified by a SynergyTM HT Multi-Detection Microplate Reader (Bio-Tek® Instruments, Winooski, VT).
