# Supplementary material for: Metabolic Signatures of Extreme Longevity in Northern Italian Centenarians Reveal a Complex Remodeling of Lipids, Amino Acids, and Gut Microbiota Metabolism
Source: PLoS One. 2013 Mar 6;8(3):e56564. doi: 10.1371/journal.pone.0056564 (PMC3590212; doi:10.1371/journal.pone.0056564)
Supplement: Text S3 — Sample preparation for 1H NMR Spectroscopy. (DOCX) [file pone.0056564.s020.docx]

**Text S3**

1ml of urine samples were dried in a freeze drying apparatus (Freeze-Dryer Fisher Scientific) and adjusted to pH 6.8 using 580 μL of a phosphate buffer solution (KH_2_PO_4_, final concentration of 0.2 M) containing 1 mM of sodium 3- trimethylsilyl)-[2,2,3,3-2H4]-1-propionate (TSP), and introduced into 5 mm NMR tubes. The standard spectra were acquired with a relaxation delay of 4s and a mixing time tm of 100 ms. Acquired ^1^H NMR spectra were processed using the Topspin software package (version 2.1; Bruker Biospin, Rheinstetten, Germany) and were referenced to the standard (TSP) at δ = 0.0. For statistical analysis all NMR spectra were converted into 12 K data points over the range of δ 0.4-10.0 and imported into the MATLAB software (version 7.11.0 (R2010b); The MathWorks Inc., Natick, MA) excluding the water residue (water δ=4.71-4.84). The spectra were normalized to the total sum of all intensities within the specified range.
