# Supplementary material for: Metabolic Signatures of Extreme Longevity in Northern Italian Centenarians Reveal a Complex Remodeling of Lipids, Amino Acids, and Gut Microbiota Metabolism
Source: PLoS One. 2013 Mar 6;8(3):e56564. doi: 10.1371/journal.pone.0056564 (PMC3590212; doi:10.1371/journal.pone.0056564)
Supplement: Text S4 — Validation of biological aging metabolic signatures in Down Syndrome (DS) individuals. (DOCX) [file pone.0056564.s021.docx]

**Text S4**

To validate the reliability/robustness of the six putative metabolic signature of aging (Trp, LPC 18:2, LPC 20:4, PC 32:0, SM 24:1, SM 16:0) we implemented targeted LC-MS/MS metabonomics approach on serum samples from DS individuals (53 subjects, average age/mean age 28.3), an accepted model of premature aging [15].While in DS, compared to age matched young control individuals (21 subjects average/mean age 30.9), levels of PC-0 32:0, SM 24:1, and SM 16:0 did not statistically changed, we noted decreased concentration of Trp, LPC 18:2, LPC 20:4 with their levels closely matching the ones of elderly and centenarian individuals (Figure S1, Table S15). We propose that these molecules are worth further investigation and validation into other aging models and/or lifespan intervention models (i.e calorie restrictions) to fully determine their functions into the aging processes.
